# Additive Ensemble Neural Network with Constrained Weighted Quantile Loss for Probabilistic Electric-Load Forecasting

**DOI:** 10.3390/s21092979

**Published:** 2021-04-23

**Authors:** Manuel Lopez-Martin, Antonio Sanchez-Esguevillas, Luis Hernandez-Callejo, Juan Ignacio Arribas, Belen Carro

**Affiliations:** 1 Department of TSyCeIT, ETSIT, University of Valladolid, Paseo de Belén 15, 47011 Valladolid, Spain; antoniojavier.sanchez@uva.es (A.S.-E.); jarribas@tel.uva.es (J.I.A.); belcar@tel.uva.es (B.C.); 2 Department of EiFAB, Campus Universitario Duques de Soria, University of Valladolid, 42004 Soria, Spain; luis.hernandez.callejo@uva.es; 3 Castilla-Leon Neuroscience Institute, University of Salamanca, 37007 Salamanca, Spain

**Keywords:** short and medium-term electric-load forecasting, quantile forecasting, deep learning, machine learning, deep learning additive ensemble model

## Abstract

This work proposes a quantile regression neural network based on a novel constrained weighted quantile loss (CWQLoss) and its application to probabilistic short and medium-term electric-load forecasting of special interest for smart grids operations. The method allows any point forecast neural network based on a multivariate multi-output regression model to be expanded to become a quantile regression model. CWQLoss extends the pinball loss to more than one quantile by creating a weighted average for all predictions in the forecast window and across all quantiles. The pinball loss for each quantile is evaluated separately. The proposed method imposes additional constraints on the quantile values and their associated weights. It is shown that these restrictions are important to have a stable and efficient model. Quantile weights are learned end-to-end by gradient descent along with the network weights. The proposed model achieves two objectives: (a) produce probabilistic (quantile and interval) forecasts with an associated probability for the predicted target values. (b) generate point forecasts by adopting the forecast for the median (0.5 quantiles). We provide specific metrics for point and probabilistic forecasts to evaluate the results considering both objectives. A comprehensive comparison is performed between a selection of classic and advanced forecasting models with the proposed quantile forecasting model. We consider different scenarios for the duration of the forecast window (1 h, 1-day, 1-week, and 1-month), with the proposed model achieving the best results in almost all scenarios. Additionally, we show that the proposed method obtains the best results when an additive ensemble neural network is used as the base model. The experimental results are drawn from real loads of a medium-sized city in Spain.

## 1. Introduction

Electric-load forecasting aims to predict future values of electricity consumption in a specific time horizon. Load forecasting is vitally important to utilities in many areas, such as maintenance, operations, planning and reliability, and, especially for modern infrastructures, e.g., smart grids [1]. Depending on the time horizon, we have short-term (range of hours to a week), medium-term (from a week to a year), and long-term (more than a year) forecasts. Depending on the expected outputs, a point forecast provides a single forecast value as the most likely estimated value of the future load. Alternatively, a density forecast provides an estimate of the future load probability distribution either point-wise (assigning probabilities to point-forecasts) or interval-wise (quantile forecasts for predefined probabilities). The first approach to density forecasting is based on extracting probabilities from a set of forecasts [2], and the second is based on quantile regression models [3].

Achieving accurate load forecasts is difficult due to the noisy and nonlinear nature of the underlying physical model. Forecasts in this area have been historically treated with time-series statistical analysis methods, e.g., autoregressive integrated moving average (ARIMA) [4], with a clear trend towards the use of machine learning techniques [5], and with emphasis on the application of generic neural network (NN) models [6] and especially deep learning (DL) models [7,8].

Load forecasting is based on a previous aggregation of load values in discrete time intervals (time-slots), which can be seconds, minutes, hours. A forecast can be only for the value in the following time slot or for several consecutive time-slot values (multistep forecast). Similarly, the forecast can be based on a different number of previous values (predictors). These predictors are formed with a sliding-window process applied to all past values. The length of the sliding window determines the number of predictors. Finally, the information to consider from the previous time-slots (predictors) can be extended to the load values and additional information, such as date/time or weather data (multivariate forecast). Including this additional (exogenous) information as new features imposes difficulties on statistical analysis methods since only a few can cope with multivariate forecasts [9]. This creates additional opportunities for machine learning and deep learning (ML/DL) techniques that easily handle vector-valued predictors. Considering all of these alternatives as additional parameters turns the forecast problem into a challenging multivariate multi-output regression problem. This work explores the influence of these critical parameters when doing time-series forecasting: sliding-window length, multistep ahead forecast length, and number/nature of features used to characterize the information used as predictors. The influence of these parameters is combined with the different nature exposed by the forecasting models.

In this work, we propose a novel quantile forecasting neural network (QFNN) model. The model is intended for multivariate multistep forecasts, i.e., vector-valued predictors (multivariate) and multistep forecasts (multi-output or multiple forecasts). A quantile forecast for a target variable creates a prediction with an associated probability that the actual values will be smaller than the predicted value (the quantile). A quantile forecast model allows quantile and prediction interval (PI) forecasts. The latter considers a pair of upper and lower quantiles. A neural network can produce quantile forecasts when using a quantile loss [10]. There are several options for the quantile loss as variants of the pinball loss [11,12]. When multiple quantile forecasts are produced, the quantile loss will average individual quantile losses. In this work, we propose a novel quantile loss that includes a constrained weighted average of the contributions to the loss made by the different quantiles in a multi-quantile forecast.

The proposed constrained weighted quantile loss (CWQLoss) extends the pinball loss to more than one quantile by creating a weighted average for all multistep forecasts (in the forecast horizon) and across all quantiles. The pinball loss for each quantile is weighted separately. The proposed method imposes additional constraints on the quantile values and their associated weights. The imposed restrictions consist of guaranteeing symmetric quantile values around the median with their associated weights having identical values for symmetric positions around the central weight (associated with the median). These restrictions are critical for a stable and efficient forecast model, as shown by experimental results (Section 4). The quantile weights are configured as learnable parameters of the NN similar to the other network weights, allowing all model parameters to be trained in a comprehensive end-to-end manner.

The resulting QFNN with the proposed CWQLoss (CWQFNN) is arranged as an extension to any NN that produces a point forecast by adding an additional layer that transforms the point forecast into multi-quantile forecasts. Therefore, the proposed model consists of (a) a point-forecast NN architecture that serves as a base model, (b) an additional layer composed of several fully connected linear layers (one for each generated quantile), (c) an end-to-end training of the resulting NN using the CWQLoss. The only requirement for a base model is to be trainable end-to-end by gradient descent and support the addition of a final layer in both the training and prediction stages. Thus, we have considered as base models several configurations of 1D and 2D convolutional neural networks (CNN) [13,14], long short-term memory (LSTM) [15] networks and their combination, as well as several additive ensembles (AE) deep learning models especially suitable for time-series forecasting [9,16]. We do not include sequence-to-sequence (Seq2seq) models as a base model since the forward pass for the training, and test stages are different with added complexity for the proposed extension.

In this work, we apply the CWQFNN architecture to short and medium-term load forecast (SMTLF), considering forecast horizons of 1 h, 1-day, 1-week and 1-month, with different numbers of predictors (Section 3.1) and with an aggregation time-slot of 1 h. We obtain the experimental results by applying the different models to a real dataset of power consumption from a Spanish utility for the province capital of Soria (Spain). This dataset has been extensively studied previously [17].

We provide a comprehensive comparison between CWQFNN and a significant number of state-of-the-art (SOTA) data-driven forecasting models, some of them widely applied to time-series forecasting and others novel or rarely applied to SMTLF, such as (a) classic machine learning (ML) models, e.g., linear regression and random forest [18,19,20,21], (b) multilayer perceptron [5], (c) deep learning models based on separate CNN and recurrent neural networks (RNN) [19,20], (d) dynamic mode decomposition (DMD) [22,23,24], (e) deep learning (DL) models based on specific combinations of CNN and RNN [25,26], (f) sequence-to-sequence (Seq2seq) models with and without soft attention [27,28,29], and (g) deep learning additive ensemble models especially targeted for time-series forecasting [9].

We have not considered time-series statistical analysis methods (e.g., ARIMA…) because they produce a model for each specific sequence of past values, i.e., the training of the model is based on a specific input sequence, which is problematic when the objective is to have a unique model that can be used to forecast any time-series from a training dataset. Furthermore, the extension of these models to vector autoregression or multivariate scenarios produces very complex models (e.g., VARIMA, VARMAX...). These types of models are called local models [6], while our interest is in global models that consist of a single model applied to the entire population of time series in our dataset. This situation is different from classic ML models, e.g., linear regression and random forest, which are global models, but produce a single output, requiring to have as many models as output values (forecast horizon length).

Considering the difficulties in evaluating the prediction performance associated with point and quantile load forecast, we have obtained six metrics to assess the performance of point forecasts and ten metrics for quantile forecasts. The point-forecast metrics provided are median absolute error (MAD), relative root-mean-square error (RRMSE) and symmetric mean absolute percentage error (sMAPE). We also provide the evolution of these metrics under different parameter values, such as the time-ahead forecast interval and the sliding-window length. The probabilistic forecast metrics considered are quantile score (QS), Winkler score (WS), sharpness and absolute average coverage error (AACE) for two central prediction intervals (PI) with probabilities of 98% and 50%.

It is worth noting the excellent results of the additive ensemble (AE) deep learning models [9,16,30] and how they excel in average results and in longer-term (most difficult) forecasts. The good behavior of deep ensembles has lately attracted considerable attention from different points of view. The best results of a deep ensemble are related to a better exploration in the solution space due to the independent random initialization of each element of the ensemble [31], in line with other studies that connect the importance of a rich set of random initializations with the behavior of deep learning models [32]. Deep ensembles can also improve uncertainty estimates for samples outside the expected data distribution [33]. This work contributes to providing additional results that confirm the good behavior of deep ensembles under an additional perspective provided by quantile forecasting. In previous works [9,30], AE has been applied to time-series forecasts with a panel data structure (a list of entities, each with an associated time-series). In contrast, this work applies it to a time-series for a single entity (a single utility) with different requirements for data preparation and the validation/testing process.

The main **advantages** of this work over related solutions based on quantile loss are: (a) Compared to loss-unweighted approaches, CWQLoss obtains the best point and quantile forecast results than unweighted quantile loss (Section 4). Additionally, the crossover rate with CWQLoss is much smaller than with the unweighted loss. Crossover occurs when a quantile forecast for a quantile with an associated lower probability is greater than the forecast for an upper probability quantile, and its rate is the estimated probability of this happening in any forecast. To achieve a low crossover rate, CWQLoss does not require the use of complex base models or intricate loss functions; meanwhile, solutions based on the unweighted quantile loss need to add strict monotonicity constraints on the network weights plus additional model parameters as in [34] or to add complexity to the quantile loss function by including crossover errors as a regularization term as in [35]. (b) Compared to loss-weighted approaches, weighted quantile regression has been applied previously, but with quantile-weights assigned manually as in [34] or not included as part of the model parameters in neural networks as in [36,37] and, none of them proposes constrained values or are learned end-to-end by gradient descent. We show that quantile forecasts improve with a weighted quantile loss (Section 4) and particularly when the quantile-weights are constrained and are learned end-to-end along with the rest of the network weights. (c) Compared to generic quantile regression models, composite quantile regression (CQR) models are known to be robust, but complex and computationally demanding [38,39,40]. CWQLoss produces robust CQR architectures with minimal increase in base model complexity and an efficient iterative optimization method using gradient descent.

The CWQFNN architecture achieves two **objectives**: (a) produce probabilistic (quantile and interval) forecasts with an associated probability for the predicted target values; (b) generate point forecasts by adopting the forecast for the median (0.5 quantiles). We provide specific metrics for point and probabilistic forecasts to evaluate the results considering both objectives. As shown in Section 4, the proposed architecture generates excellent prediction results for different base NN models with few extra requirements in terms of computation time and added complexity. The model presents the best point-forecast results for long-term forecasts and excellent quantile-forecast results using probabilistic metrics.

The motivation of the work is to propose a novel technique that is useful for point-forecasts, which are important for the operations and planning of utilities, and contribute to the availability of probabilistic forecasts as a valuable tool to identify new applications, such as (a) detection of anomalous consumption patterns due to excessive deviations from prediction intervals, which can be used as alarms for security or fraud situations and, (b) what-if simulations for non-standard load scenarios and their consequences. Legacy electrical grids are evolving to so-called smart grids, where different parts of the grid are being modernized thanks to information and communication technologies (ICT) and IoT (Internet of things). Despite its many advantages, one of its main risks is related to cybersecurity and fraud attacks [41], and any initiative to help in this area could be valuable.

The **contributions** of this work are: (a) propose a novel QFNN architecture that includes a new quantile loss that allows extending a regression NN, acting as a base model, to become a quantile forecasting model; (b) provide a new weighted quantile loss that is based on specific constraints easily incorporated into the network model, allowing and end-to-end training of all model parameters by gradient descent; (c) propose an architecture (CWQFNN) that does not require transforming the base model [34] or adding complex extensions to the loss function [35] to ensure efficient quantile forecasts with a small crossover rate; (d) present a thorough analysis and comparison between CWQFNN and a significant number of alternative methods with a special emphasis on novel methods, e.g., additive ensemble deep learning, DMD, Seq2seq and combinations of CNN/RNN models; (e) show the excellent performance results obtained by CWQFNN in general and its particular good combination with an additive ensemble (AE) deep learning base model; (f) apply all the models to a previously well-studied dataset of real electricity consumption, allowing comparisons to be made on a single dataset in a homogeneous and structured way, which allows comparing results and drawing conclusions on a common basis; (g) include the influence of important parameters in the study, i.e., sliding-window length, k-step ahead forecast length, and a number of features associated with the time-slots; (h) present the best groups of models according to different forecast objectives; (i) apply for the first time to SMTLF, as far as we know, an AE deep learning model [9] and extend it to the particular needs of SMTLF.

The paper is organized as follows: Section 2 summarizes previous works. Section 3 describes the dataset and models employed. Section 4 details the results, and Section 5 presents the conclusions.

## 2. Related Works

We will present related works considering the applied methods and global review studies. The presentation will focus more on adopted methods and processes than on performance metric comparison since the diversity of datasets, the difference in load magnitudes, the differences in the implementation of the metrics and the various test/validation procedures make it very difficult to perform a homogeneous comparison of results. We will focus on related works corresponding to the alternative models used to compare the results obtained by CWQFNN, as well as works related to quantile forecasting applied to SMTLF:

- **Review works:** The work in [5] presents a comprehensive review of the techniques used to forecast electricity demand, analyzing the different types of forecasts, parameters affected, techniques used, together with a literature review and a taxonomy of the main variables involved in the problem. The work in [19] presents a detailed review of recent literature and techniques applied for building energy consumption modeling and forecasting.
- **Quantile forecasting applied to SMTLF:** The work in [10] presents theoretical bases on the effectiveness of the pinball loss function to achieve quantile estimations. A comparison of quantile regression techniques for weather forecasting is provided in [42] with a recommendation to use ensemble models. A gradient descent algorithm for quantile regression is proposed in [12]. The work proposes a special function to smooth the pinball loss. The technique is extended to a boosted quantile regression algorithm, and the results are obtained with simulated datasets. There are several works presenting probabilistic forecasting neural networks for load forecasting. In [43], a smoothed quantile loss with a CNN network is used to build a multi-quantile forecast estimator. The pinball loss is smoothed with a log-cosh function. The model is applied to residence load forecasting. A similar model is proposed in [44] with an NN based on ResNet with skip connections. The pinball loss is not smoothed. The work analyzes the impact of the network depth and the skip connections. The dataset used is the open-source GEFcom2014. In the same line of work, [45] obtains quantile forecasts with an NN based on an LSTM network. A Huber smooth function is applied to the pinball loss. The work presents results for residential and small businesses load forecasting using a public data set. The same smooth pinball loss proposed in [12] is used in [11] for quantile forecast of energy consumption using the GEFcom2014 dataset. To reduce quantile crossover, they propose a special weight initialization of the neural network. In [35] the quantile loss is regularized with an additional term to take into account the crossover quantile errors. The dataset used is also GEFcom2014. All the previously mentioned works apply variants of the quantile forecasting model, including neural networks, but none propose a constrained weighted quantile loss fully incorporated as learnable parameters in the network architecture and capable of extending any point-forecast NN into a quantile forecast model.
- **Dynamic mode decomposition (DMD) applied to SMTLF:** Considering related works corresponding to the alternative models used as comparison models for the CWQFNN (Section 4), there is a growing current interest in the application of dynamical systems analysis tools based on reduced-order models and, in particular, in the use of dynamic mode decomposition to SMTLF. The work in [24] provides a DMD study applied to electric load data from a utility operator in Queensland, Australia. They show better performance results using DMD vs. time-series autoregressive models. The forecasting is made for the following day using the data from the previous 4 days as predictors, presenting the best result for mean absolute percentage error (MAPE) for one-day ahead forecasting of 2.13. A similar application of DMD is done in [46] but applying DMD to predict forecast errors followed by an extreme value constraint method to further correct the forecasts. The algorithm is applied to actual load demand data from the grid in Tianjin, China, and the results obtained with DMD are compared with a series of additional techniques (autoregressive moving average, neural networks, support vector machines, extreme learning machines...). According to the authors, the proposed method shows greater accuracy and stability than alternative ones, with a best average root-mean-squared error (RMSE) of 476.17. In [47], the authors employ an empirical mode decomposition technique to extract different modes from the load signal and apply an independent deep belief network for each mode prediction, with a subsequent aggregation of results (ensemble) to obtain the final load forecast.
- **Classic machine learning models applied to SMTLF:** A substantial number of works have presented several classic machine learning models for SMTLF. A feed-forward neural network (FF-NN) is used in [48] to forecast the electricity consumption for residential buildings for the next 24 h. Results are compared with other models, including GTB and RF, selecting the best model at each forecast iteration. The best average result for the different test iterations is obtained for the neural network (NN) with an RMSE of 2.48. The work in [49] presents a theoretical review of the most commonly used ML methods for short-term load forecast (STLF), including NN and support vector for regression. Time-series statistical analysis models for SMTLF are discussed in detail in [4] with forecasts at an hour interval applied to load data from the Electric Reliability Council of Texas (ERCOT). The present results are applying ARIMA and seasonal autoregressive integrated moving average (SARIMA) models achieving an average MAPE between 4.36% to 12.41%. More classic ensemble techniques for forecasting electricity consumption in office buildings are investigated in [50], comparing gradient tree boosting (GTB), random forests (RF) and a specifically adapted Adaboost model that presents the best results.
- **Sequence to sequence (Seq2seq) models applied to SMTLF:** Seq2seq architectures that originated in the field of natural language processing (NLP) have been applied in recent works to STLF. Authors in [51] apply different Seq2seq architectures, comparing them with other DL models based on recurrent and convolutional layers. The models are applied to two different datasets (scenarios), one for an Individual household electric power consumption data set (IHEPC) and the other for the GEFCom2014 public dataset. The best results (RMSE between 17.2 and 0.75 depending on the scenario) are obtained with convolutional and recurrent architectures and deep neural networks with dense layers. Considering average results, the Seq2seq models do not provide the best results. The conclusions obtained in this work are consistent with the results obtained by the present study. A similar study is presented in [52], where research is conducted comparing a Seq2seq model (with and without attention) with alternative DL models based exclusively on different types of recurrent networks, such as long short-term memory networks (LSTM) and gated recurrent unit (GRU). In this case, the Seq2seq model presents the best results for short-term forecasting, also following the results obtained in the present work. A generic Seq2seq with a specific attention mechanism is proposed in [53] for multivariate time-series forecasting.
- **Deep learning models applied to SMTLF:** The work in [54] introduces a deep learning architecture based on an ensemble of convolutional blocks acting on segregated subsets of the input data. The model is applied for day-ahead forecasting of individual residential loads with data obtained from a smart metering electricity customer behavior trial (CBTs) in Ireland. The work focuses on achieving low training time and high accuracy, the proposed model being the best in both aspects with a mean absolute error (MAE) of 0.3469. Authors in [55] present an analysis of the influence of the number of layers, activation functions and optimization methods using neural networks to predict the Hellenic energy consumption. The work in [56] incorporates a wavelet denoising algorithm to a neural network for the short-term load forecast of the Bulgarian power system grid, showing that wavelet denoising improves the load signal quality and overall forecast performance.
- **Models applied to the same dataset:** Using the same dataset proposed for this work, [17] presents an NN model that works on a 24 h day-ahead forecasting of electric loads previously aggregated into clusters by consumption patterns. The patterns are obtained with a self-organizing map (SOM) followed by a k-means clustering algorithm.
- **Application to cybersecurity:** The impact of cybersecurity attacks on smart grids is well-known [57], these attacks can be addressed with intrusion detection tools, but there is a growing interest in identifying these attacks using indicators of indirect effects, such as deviations from normal consumption or customer revenues. In these alternative approaches, the application of accurate forecasting models is crucial. The detection of abnormalities in load consumption patterns to identify energy theft or other types of attacks is studied in [58], based on a hierarchical clustering and decision trees classification. A similar approach is presented in [59], which also uses a decision tree algorithm without prior clustering.
- **Fuzzy methods with probabilistic forecasts:** Several works explore using fuzzy methods concerning probabilistic forecasts, either as alternative methods [60] or as prediction evaluation methods [61], as well as recent advances in fuzzy methods [62,63].

## 3. Materials and Methods

In this section, we provide details of the dataset used for the experiments and the forecasting models considered for this work. The electricity consumption dataset and the proposed models are presented in Section 3.1 and Section 3.2, respectively.

### 3.1. Selected Dataset

The dataset used for this work corresponds to real data from a Spanish utility and is formed by historical electricity consumption over three years, from the province capital of Soria (Castilla y Leon, Spain). The logged consumptions vary in the range between 7 to 39 MW, which is much lower than observed in large and aggregated environments, and with a load curve sharing similar features to that of a microgrid [17].

**Figure 1:**
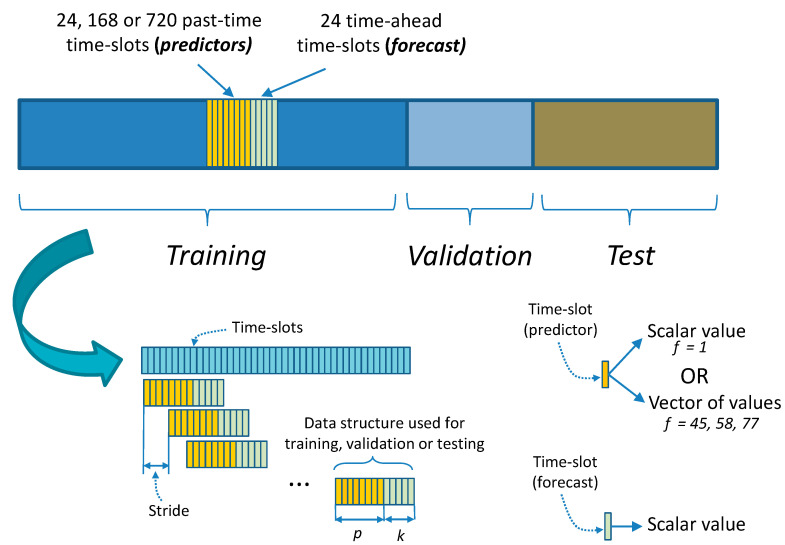
Structure of the dataset used for training and evaluation of the forecasting models. The original dataset is divided into training/validation/test sets. A sliding window is applied across the entire dataset to create the data structures used for training/validation/test. Different values of p (number of time-slot predictors) and k (number of time-slot forecast) can be set depending on the model configuration.

Consumption data have been aggregated in time-slots of one hour, adding other additional variables related to date/time and weather as additional features. The total number of hours of aggregate consumption is 26,302 h. The date/time features considered month, time, day of the week and weekend indicators. The weather features considered are mean and standard deviations for atmospheric pressure, wind speed, wind direction (degrees), humidity and solar radiation. All date/time features have been treated as categorical variables and have been one-hot encoded. Weather features are continuous features. All continuous features, including the electricity load, have been scaled in the range [0–1]. After coding and scaling data, we have obtained four different sets of features: (a) 1 feature, which corresponds to the electricity load; (b) 45 features, corresponding to the date/time (day of week, weekend, hour, month) and load features; (c) 57 features, corresponding to the date/time, weather and load features; and (d) 76 features, corresponding to the date/time and load features plus the one-hot encoded day of the month. Experiments performed with different feature sets have been reported separately in Section 4. The number of features associated with different feature sets is referred to by the symbol f (Figure 1). To get a manageable number of results, we have used only the feature sets with f equal to 1 and 45. The weather features, when applied, did not provide a noticeable improvement and could hinder the possibility of transferring the results to other datasets since weather data cannot always be obtained or could be different from those used in this work. Similarly, the inclusion of the day of the month did not provide improvements in different experiments. It was not considered since, in addition, a greater number of features produces additional difficulties in training the models without adding additional advantages in this case.

To prepare the dataset to be used by the different models, it is necessary to segregate it into an array of data structures used during training, validation, and test. Figure 1 presents the process to create these data structures, which are formed by a sequence of data associated with the time-slots used as predictors and the number of time-slots to be predicted. Associated with the time-slots used as predictors, we use the different feature sets described above. The predicted values always correspond to the electricity load (a scalar). The data structures (Figure 1) are created by applying a sliding window to the entire data set ordered in time. The stride applied to the advance of the sliding window is presented in Figure 1 and has been considered with a value of 1 for all the experiments. The symbol p (Figure 1) will refer to the number of time-slots used as predictors (sliding window length), and the symbol k (Figure 1) will refer to the number of predicted time-slots (k-step ahead forecast length). Once these data structures are created, we separate them into two initial groups used as training and test data. Separation is carried out along the time variable, with the first 80% of the data as training data and the last 20% as test data. Furthermore, for all DL, Seq2seq and ensemble models, the training data are additionally subdivided into 20% validation data and the remainder as final training data. The validation data are used to assess model performance during training.

**Figure A1:**
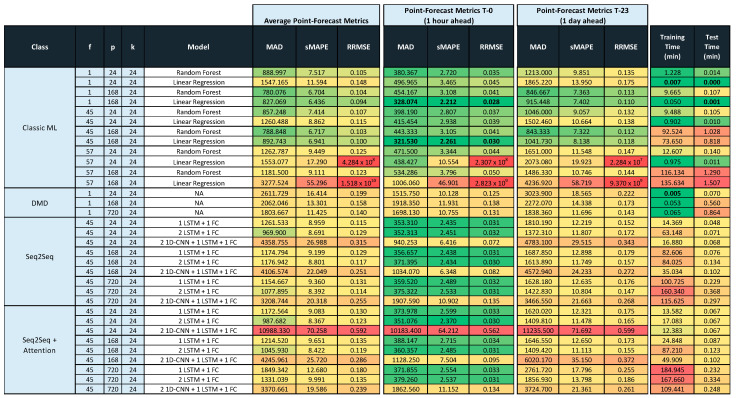

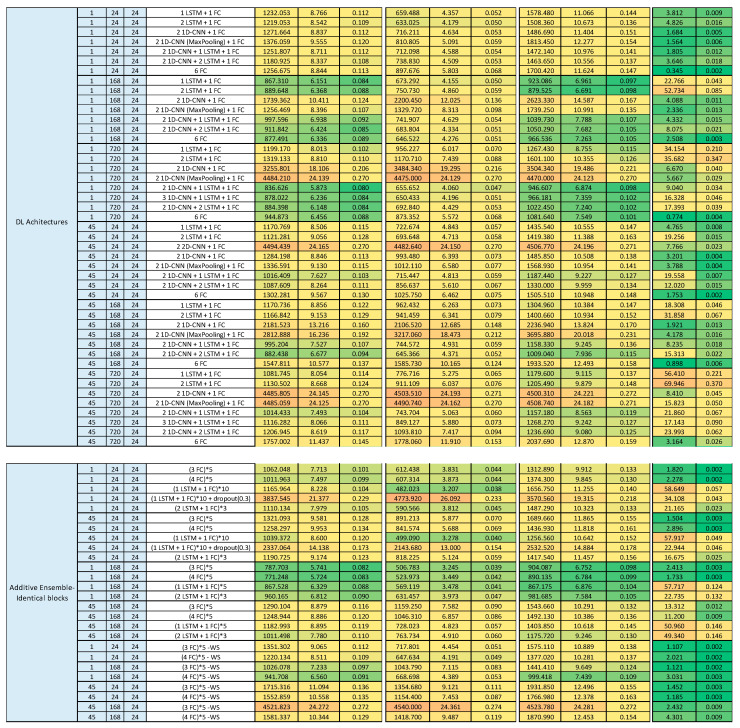

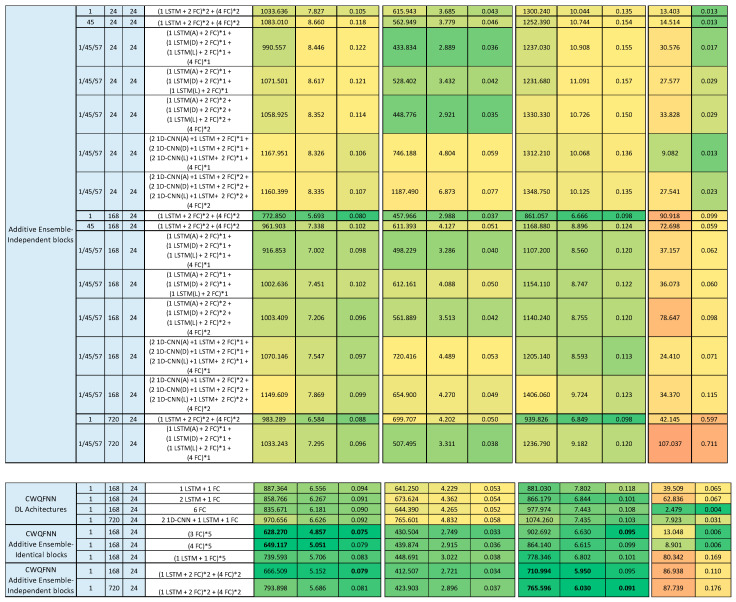
Point forecast performance metrics for the average, first (T-0) and last (T-23) predicted time-slots using all models. This table is limited to a forecast time horizon of 24 h (k = 24). The average is obtained along the 24 predicted values (k = 24). Different number of predictors (p) and network architectures are considered for several values per predictor (f). Table is color-coded (column-wise) with a green–red palette corresponding to best-worst results, respectively. The best two values per column are highlighted in bold. Section 3.2 and Section 4 provide how to interpret the description of the models and their groups. The last two columns provide the training and test times for all models. This table shows the complete set of models used to compare results for k = 24. The results presented in Section 4 are the most representative results obtained in this table. The models considered most representative for k  = 24 are those considered to compare the models for k  = 168 and 720.

**Figure A2:**
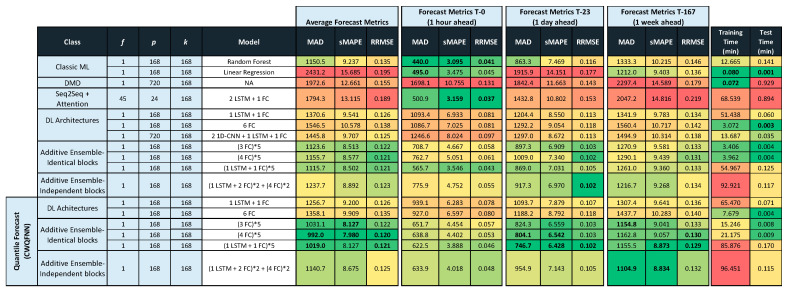
Point forecast performance metrics for the first (T-0), 23rd (T-23) and last (T-167) hour forecast and average forecast over a 168-h (1 week) forecast time horizon (k = 168). Results for a selection of best models for each group of models. Table is color-coded (column-wise) with a green–red palette corresponding to best-worst results, respectively. The best two values per column are highlighted in bold. The last two columns provide the training and test times for all models.

**Figure A3:**
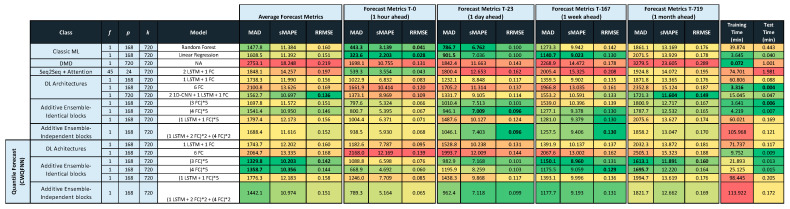
Point forecast performance metrics for the first (T-0), 23rd (T-23), 167th (T-167) and last (T-719) hour forecast and average forecast over a 720-h (1 month) forecast time horizon (k = 720). Results for a selection of best models for each group of models. Table is color-coded (column-wise) with a green–red palette corresponding to best-worst results, respectively. The best two values per column are highlighted in bold. The last two columns provide the training and test times for all models.

Depending on the number assigned to f, *p* and *k* (Figure 1), a different dataset will be created from the original data. The range of values assigned to *f*, *p* and *k* are: (a) *k* will be 24 (1-day forecast horizon), 168 (1-week horizon) or 720 (1-month horizon), (b) *f* will be 1 or 45 and, (c) *p* will be 24 (using the previous day of data -24 h), 168 (previous week of data-168 h) or 720 (previous month of data-720 h). Each combination of these values (f*, p, k*) will be assigned to different groups of results in Section 4 and Appendix A (Figure A1, Figure A2 and Figure A3).

**Figure 2:**
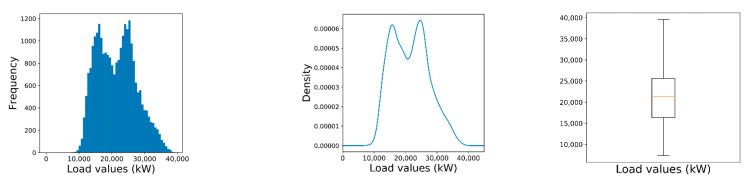
Histogram (**left**), density (**center**), and boxplot (**right**) for the distribution of load values. In the charts, the load values are in kilowatts (kW), the frequency identifies the number of times a certain load appears in the dataset, and the density is the normalized frequency.

Figure 2 presents the distribution of electrical power consumption reported in the dataset. We observe a bimodal distribution with a total average value of 21,478.2 kilowatts (kW) and a standard deviation of 5949.5 kW. The two modal values are presented around 15,000 and 25,000 kW, with a value range between 7370 kW and 39,550 kW. Figure 2 provides the histogram, density, and boxplot for the distribution of load values to be predicted.

**Figure 3:**
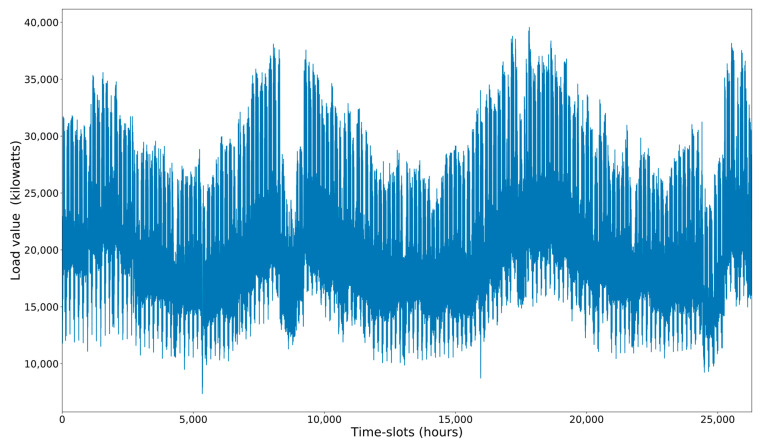
Load values over time for the entire dataset (spanning 3 years). Load values are given for time-slots of 1 h. The load values vary from 7,370 kW to 39,550 kW with a time span of 26,302 h.

Figure 3 provides an additional view of load values over time for the entire dataset (spanning 3 years). We can see that the values have a clear annual periodicity. The values also have a strong daily and weekly periodicity.

### 3.2. Models Description

In this section, we present the different models used in our research, describing their main characteristics and pointing out useful references. Our main interest will be to describe in detail the proposed CWQFNN architecture. The other models serve as alternatives for comparing results and will be briefly described with references to the original works. We have grouped the models for similarity and to facilitate the subsequent presentation of the results (Section 4). The groups considered are the following:

Classic machine learning models: Machine learning models are widely used in STLF, with most models already tested in some aspects of STLF. In this study, we have focused on two models: linear regression and random forest. These models combine their good performance and robust behavior without requiring exhaustive hyperparameters tuning.

Dynamic mode decomposition (DMD) models: These models are novel applications of linear transformations that attempt to approximate the latent non-linear model of a system by a best linear approximation [23]. They have been used very effectively in various fields (fluid dynamics, finance, etc.) for system identification and forecasting [22,64]. In addition to providing good regression estimators, they also give information about the fundamental behavior of the underlying system. The methods made available by DMD are a recent focus of interest in STLF [24].

Seq2seq models: The sequence-to-sequence models were initially used in NLP, but their applicability has spread to almost any time-series forecasting problem. Until now, these models have been little applied to STLF, and their results have been good for very short-term forecasts [52].

Deep learning models: As already mentioned, deep learning models are currently the main trend in STLF. We have applied various configurations of convolutional neural networks (CNN) and long short-term memory (LSTM) networks, a type of recurrent neural network (RNN). The combination of CNN and LSTM networks has provided some of the best results, following the results obtained in other works applying the same configurations to other fields (network traffic analysis, the video quality of experience, etc.) [25,26].

Additive ensemble neural network models: It is well-known that aggregating the capabilities of various estimators can increase their effectiveness in reducing errors and avoiding overfitting. There are several aggregation strategies, and boosting is one of the most important obtaining state-of-the-art estimators. Bringing together boosting and deep learning has shown very good results in other classification/regression problems [9,16]. The additive ensemble models considered in this work will follow the gaNet architecture [9], a deep learning boosting ensemble model specifically intended for time-series forecasting.

Quantile forecasting neural network (QFNN) models: In this group, we will present in detail the CWQFNN architecture based on the constrained weighted quantile loss (CWQLoss) that generates multi-quantile forecasts. The CWQLoss allows extending a regression NN, acting as a base model, to become a quantile forecasting model.

Assuming that we have a time-series of vector-valued predictors $\left\{x_{t-p},..,x_{t}\right\}$ of length p that ends at a generic time t. As presented in Section 3.1, a vector-valued predictor at any specific time contains features about the 1 h interval starting at that time. These features are the elements of the vector, that is, for one predictor $x_{t}$: $x_{t}={\left(v_{t,j}\right)}_{j=1}^{f}$, f is the number of features (components) of the vector-valued predictor $x_{t}$, and $v_{t,j}$ is the j feature of the predictor. The features included in the vector contain, as a minimum, the electric load for that 1 h interval. Additional features are day/hour identifiers or weather statistics for the 1 h period. These additional features can be included or not depending on different training configurations.

With these predictors, the goal is to provide a multivariate multiple regression model (forecast model) [65] that generates a time-series forecast with scalar values of length k starting at time t+1. These scalar values correspond to the load forecast (target variable) for times [t+1,..,t+k]. The parameters f, p and k have an impact on the forecast and are considered separately in the results of the models given in Section 4.

A point forecast corresponds to a single forecast associated with the conditional expected value of the target variable conditioned on the predictor’s value. Instead of the mean (expected value), other reference statistics can be considered for the target variable, such as the median or other quantile associated with the probability distribution of the target variable. In our case, a quantile forecast (probabilistic forecast) for a time *t* and quantile’s probability q must provide k forecast values for times t+1 to t+k with probability q of having their ground-truth values smaller than the forecast values, i.e., a quantile forecast for q=0.75 should have its ground-truth value smaller than the forecast with probability 0.75. Quantile forecasting allows you to create confidence intervals (CI) when two quantiles are used to define a central prediction interval (PI) with a probability that the actual values are in it. The probability assigned to the CI is the difference between the defining quantiles. For example, the quantile forecasts for quantiles 0.1 and 0.9 will define a PI with an associated probability of 0.8, i.e., 80% of the actual values are expected to be in this PI.

**Figure 4:**
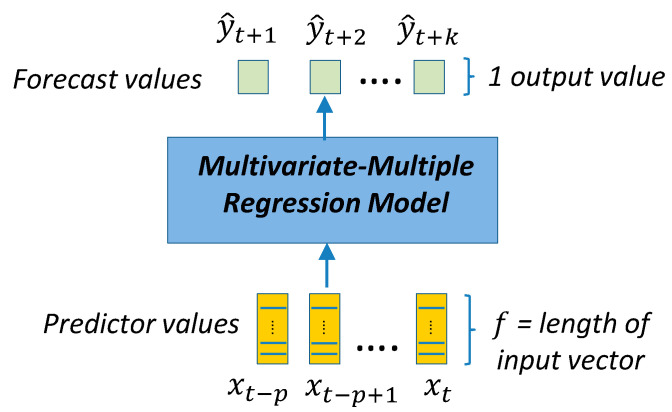
General architecture for the multivariate-multiple regression model used as the reference for all models.

**Figure 5:**
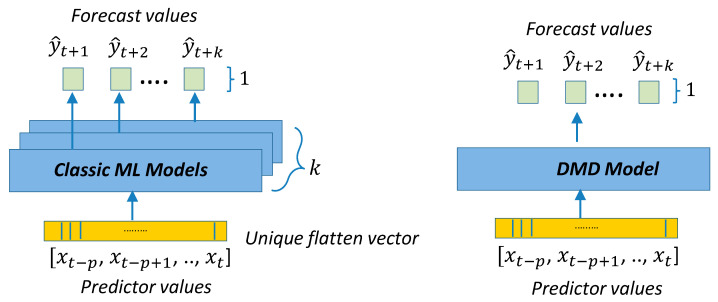
General architecture of the classic machine learning (ML) and dynamic mode decomposition (DMD) models. ML models produce a single output, hence the need to generate one model per output. DMD models support multiple outputs (k > 1).

**Figure 6:**
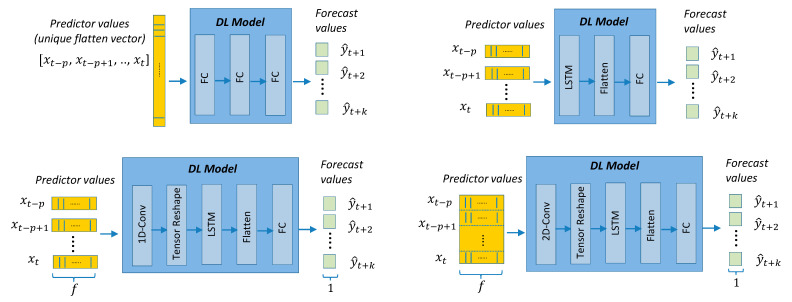
General architecture of the deep-learning (DL) models, including 1D convolutional (1D-conv), 2D convolutional (2D-conv), long short-term memory (LSTM) and fully connected (FC) layers.

**Figure 7:**
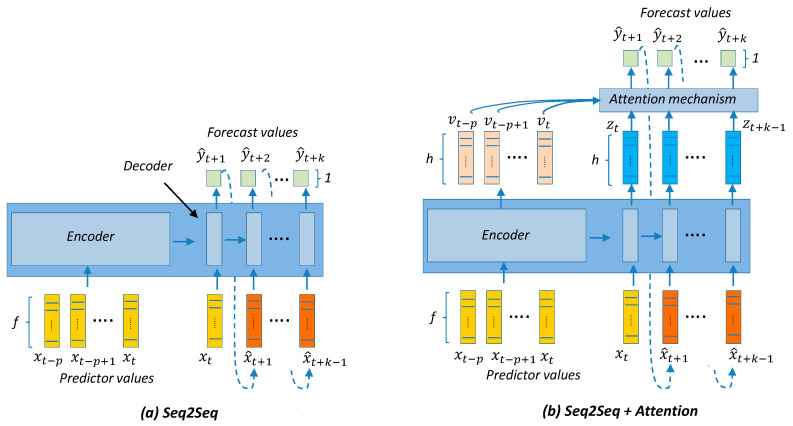
General architecture of the Seq2seq and Seq2seq+ attention models. The architecture is formed by two blocks: encoder and decoder. The encoder creates a latent representation for the inputs, and the decoder creates the output in an iterative process from the encoder’s output and the previously made forecast. The attention mechanism allows the decoder’s output to be weighted with the most similar intermediate outputs of the encoder.

**Figure 8:**
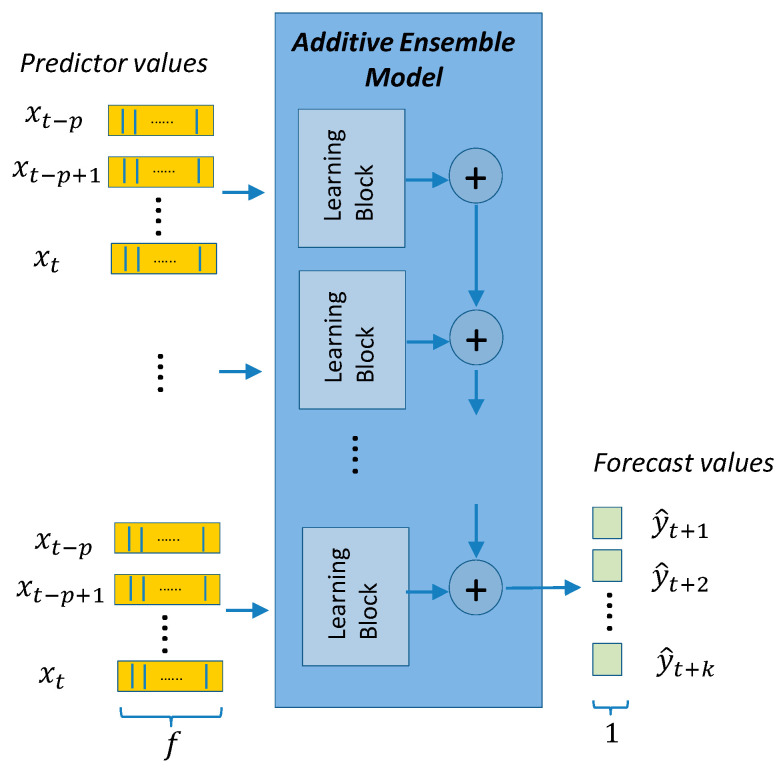
General architecture of the additive ensemble (AE) deep learning model. The final output is generated by the output aggregation of several DL blocks applied to the same inputs. The blocks can have identical structures (even sharing weights) or independent structures (each one different).

**Figure 9:**
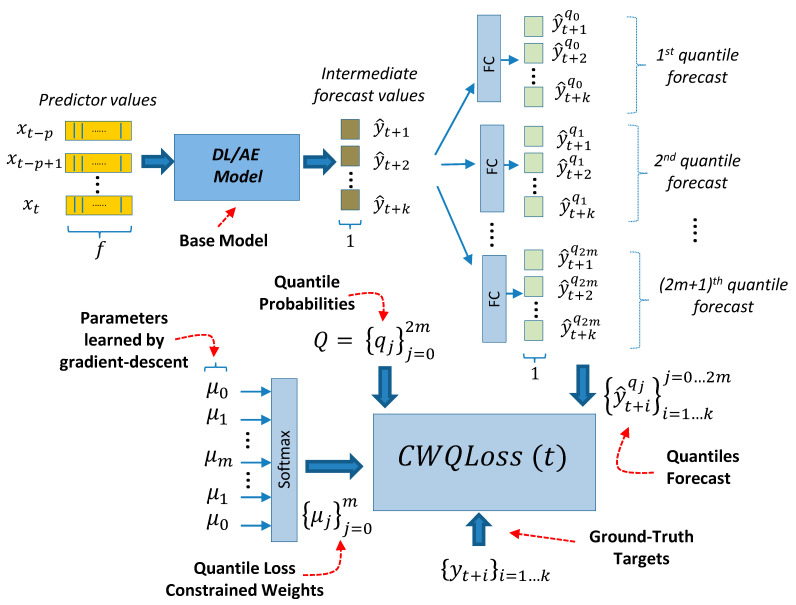
Architecture of the CWQFNN model. It is a generic architecture to extend the regression models shown in Figure 6 and Figure 8 (named as the base model). A deep learning (DL) or additive ensemble (AE) model serves as the base model, followed by an extension formed by a series of fully connected (FC) parallel layers. This extension replaces point forecasts with probabilistic forecasts (quantile forecasts). As a minimum, the forecast for the median value (0.5 quantiles) is always included and used as the final point forecast. The rest of the quantiles are constrained to have symmetric probabilities around the median, e.g., {0.01, 0.25, 0.5, 0.75, 0.99}. A new loss is defined: constrained weighted quantile loss (CWQLoss), where each quantile prediction is individually weighted also using symmetry-constrained weights (Equation (2)).

The models presented in this section are divided into two categories: (a) point-forecast models shown in Figure 4, Figure 5, Figure 6, Figure 7 and Figure 8, and (b) quantile-forecast models shown in Figure 9.

Figure 4 shows the reference to the generic regression algorithm needed to transform the input sequence of p predictors into the output forecast sequence of length k. This generic structure will be the framework used by all point-forecast models used in this work. Figure 5 presents the framework for the classic ML and DMD models. Figure 6 presents the DL architectures used in this study. Figure 7 presents the details for the Seq2seq model with and without attention. Figure 8 presents the details for the additive ensemble architectures [9], which are deep learning ensemble configurations based on gradient boosting principles and particularly suitable for time-series forecasting. Figure 4, Figure 5, Figure 6, Figure 7 and Figure 8 present a schematic view of the models, emphasizing the inputs received and the generated outputs. We can observe how the input formats depend on the type of model. The ML and DMD models expect a sequence of scalar values (longitudinal data) as input; the way to transform the input data for these models is to flatten the vectors over all time-steps. The DL models can receive vector-valued inputs, i.e., both LSTM [52] and 1D/2D CNN [51] models can receive a vector-valued sequence (with length p) where each timestep is represented by a vector of values. When the first layer of the DL model is an FC layer, the input must be formatted as a vector (flattened), and when the first layer is a 2D-convolutional (2D-conv) layer, the data must be formatted as a matrix by packing the predictors (in columns) for all past time-steps (in rows). The input format of the additive ensemble model (Figure 7) depends on the architecture of the learning blocks; these learning blocks may or may not be identical, and their architecture may be any of the architectures shown in Figure 6.

The classic ML models used in this work: linear regression (LR) and random forest (RF) [19], are two well-known models, robust and not prone to overfitting and easy to train. These models present excellent training and prediction times and good forecasting metrics for very short-term forecasts (Section 4). However, they show much larger operational times for very large p and f values, which causes that the inputs to the ML models (a flattened vector) to be extremely large, leading to memory allocation problems and indirectly greater computational times. Both LR and RF handle single-output forecasts, which means that we need an independent estimator for each of the k-step ahead forecasts, in our case 24, 168 or 720 estimators. Each of these estimators also requires independent training. It is interesting that even with an independent estimator per output, for long-term forecasts, other multi-output models can produce better results for these distant forecasts (Section 4). The capacity of this alternative model to account for possible correlations between the different outputs can explain this behavior since, in the case of the ML models, each estimator has no knowledge about the other outputs.

Dynamic mode decomposition (DMD) [23] is a linear reduced-order model (ROMs) that provides low-rank approximations of complex dynamical systems. It is a data-driven model that, in addition to making predictions about the future system behavior, is also capable of discovering the temporal modes of the system (frequencies of oscillation with its growth or decay rate) by extracting the main eigenvalues and eigenvectors of the linear mapping that transforms a snapshot of the output sequence of the system into itself advanced one time-step. It is a method that has received attention in recent times and is discussed compared to DL models.

The DL models considered follow four configurations, depicted in Figure 6: (a) simple multilayer perceptron with several fully connected (FC) layers, (b) recurrent networks formed by one LSTM layer or two stacked LSTM layers plus a final FC layer, (c) networks formed by a combination of one to three 1D-convolutional (1D-conv) layers (common for time-series) followed by one or two LSTM layers and a final FC layer, (d) combinations of 2D-convolutional (2D-conv) layers followed by LSTM layers and a final FC layer. The last two configurations have shown very good classification and regression performance in other fields [25,26] and other time-series forecasting problems [9]. For the 2D-conv layers, it is necessary to transform the input sequence of vectors into a matrix that is interpreted as a pseudo-image, following the approach in [26]. Contrary to the good performance of this approach in other fields, in this case, the results obtained by the architecture: 2D-CNN+LSTM have not been as good as expected (Section 4 and Appendix A (Figure A1, Figure A2 and Figure A3)).

The output from the LSTM layers is a multidimensional array (2 dimensional) that needs to be flattened before serving as input to an FC layer (Figure 6). Similarly, the outputs from the 1D and 2D-conv layers (also multidimensional arrays) need to be transformed (tensor reshape) to the input dimensions expected by an LSTM layer. The activation function for all layers has been ReLU except for the last layer with a linear activation. The cost function used has been the mean squared error.

Sequence-to-sequence models (Seq2seq) [28] were originally intended for the classification and forecasting of time series with discrete and complex categorical predictors (text, words, etc.). Its use has become widespread in many different fields where the objective is to make a multi-output forecast based on an embedded (latent) data representation of all past information along with an iterative process that combines this latent representation with individual forecasts for each time-step ahead (Figure 7). Seq2seq models are made up of two blocks: an encoder and a decoder. The encoder has the mission of constructing the latent representation by producing a vector (embedding) that summarizes all the information contained in the input sequence. The decoder takes the vector embedding and performs the iterative process of producing forecasts one-by-one, using the forecast produced in the previous step as input for the forecast in the next step. The layers used in both the encoder and decoder blocks are similar and based on recurrent layers, in our case, LSTM layers. There is also the possibility of incorporating other additional layers before and after the recurrent layers, such as CNN layers, to perform representation learning of the input sequences and fully connected layers as final layers.

Seq2seq models can incorporate an attention mechanism [27,29] that consists of an additional layer that implements a similarity comparison between an initial forecast ($z_{t}$) and information related to past history ($v_{t}$) (p previous values used as predictors). The similarity operation can be smooth and differentiable (soft attention) or not differentiable (hard attention). We have used soft attention based on a softmax applied to the dot product of the initial forecast with all the intermediate results produced by the *p* values used as predictors. The diagram in Figure 7 shows how the attention mechanism works by applying a distance function to past history and incorporating that distance in the later stages of the forecast.

As already noted, we have not considered statistical time-series analysis methods (e.g., ARIMA) because they produce forecasts based on a specific sequence of predictors, i.e., model training is based on a specific input sequence and not in an entire dataset of training sequences, and is problematic when the goal is to have a single model that can be used to forecast any time-series from a given dataset.

The DL ensemble model used in this work follows the additive ensemble architecture in [9] that presents the gaNet model based on creating an estimator by aggregating blocks formed by small DL networks (Figure 8). All blocks are arranged in sequence, all sharing the same input. The output from the first block is aggregated to the output from the following blocks until the final output is produced. The aggregation process begins with a fixed value (usually an average of the expected results). The aggregation function used is the sum, but other functions, such as the mean or maximum value, can also be used. It is important to note that all blocks are trained together end-to-end using gradient descent. The gaNet architecture has already been extended to be used for regression [9] and classification [30] for IoT traffic analysis and forecasting. This is the first application of this architecture to SMTLF. It is formed by the aggregation of small building blocks integrated by a few CNN and/or RNN layers. This architecture can be considered as a simpler implementation of gradient boosting ideas with a single end-to-end training (instead of stage-wise), and where the DL building blocks are taking the role of decision stumps, which are the basic blocks of classic gradient boosting models. Alternatively, it can also be seen as connected to stacked models [66] and residual networks [67], with all short-cuts (layer jumps of residual networks) sharing the same inputs. In the basic gaNet model, all blocks have the same layer configuration (architecture), and all blocks in the sequence are similar but not identical, as random initialization of their weights and end-to-end training will induce different weights in each of the blocks. There are also variants on this basic configuration by allowing different block architectures and other training options. In [9], the gaNet model is presented in detail with several variants (types), considering if the blocks are all identical or not, if they share their weights or if the loss function is a unique function or is formed by adding the loss functions of the intermediate outputs. Considering all the gaNet variants, we have chosen the two most generic that we have named as: ‘additive ensemble-identical blocks’ and ‘additive ensemble-independent blocks’. An ‘additive ensemble-identical blocks’ model is made up of identical blocks where each block is formed by small DL networks consisting of 1D-CNN, LSTM and FC layers. We have also differentiated a subgroup of models where all the blocks share their weights. In this case we have blocks not only with identical architecture, but with identical weights. It is interesting to investigate this specific subgroup because they are models with very few weights that can be important to avoid overfitting. These models have a suffix (WS) at the end of their description (Section 4). An “additive ensemble-independent blocks” model has blocks separated into groups where each group can have a different architecture with all blocks in the same groups sharing the same architecture. For this model, we indicate separately the number of repetitions of identical blocks per group (Section 4). There is freedom in the number of groups and blocks per group.

The new proposed model (CWQFNN) is a generic deep learning model shown in Figure 9. It extends the regression models presented in Figure 6 and Figure 8 to provide quantile forecasts. The time-series of vector-valued features used as predictors are the entry point for any of the forecasting regression models shown in Figure 6 and Figure 8. The output from these models (a forecast of length k) ${\left\{{\hat{y}}_{j}\right\}}_{j=1}^{k}\;$ is delivered to an additional and final layer consisting of several simple FC layers with linear activations, one for each of the quantile forecasts. This final layer produces M quantile forecasts ${\left\{{\hat{y}}_{i}^{q_{j}}\right\}}_{i=1\ldots k}^{j=1\ldots M}\;$ each with a forecast horizon of length k. The extra complexity added by this last layer is minimum. Each of these final quantile forecast layers has identical input and output dimensions (k). The loss function for the model in Figure 9 is shown in Equation (2). Assuming that we obtain the quantile forecast for all quantiles with associated probabilities in a set Q. The number of elements in the set Q is represented as |Q|, where |Q|=M=2m+1, for a certain m ≥0.Then, we produce an odd number of quantile forecasts (M is an odd number), where the forecast for the median (0.5 quantiles) is always included. This median forecast is adopted as our point forecast in the results presented in Section 4.1. Therefore, the CWQFNN architecture produces two types of forecasts: (a) M quantile forecasts with an associated probability for the predicted target values, and (b) a point forecast by adopting the forecast for the median (0.5 quantiles) as the point forecast. Our goal is to create an architecture that performs well in both types of forecasts.

We define a new loss based on the pinball loss (Equation (1)). Pinball loss is defined as the maximum of the difference between the ground-truth target value (y) and its predicted value ($\hat{y}$) multiplied by either the quantile (q) value (a probability) or 1 minus that value. The new loss (CWQLoss) is defined in Equation (2). It extends the pinball loss as an average for all quantiles (1…M) and all predictions in the forecast window (1…k). As defined in Equation (2), the CWQLoss (t) is a loss for a specific time-sequence of samples of length p that ends at time t that intends to evaluate the quantile forecast errors for a forecast time horizon of length k and for M quantiles (Figure 9):

(1) $$PinBallLoss=max\left[\left(q-1\right)\left(y-\hat{y}\right),\;q\left(y-\hat{y}\right)\right]$$

(2) $$CWQLoss\;\left(t\right)=\frac{1}{k\cdot \left(2m+1\right)}\overset{2m}{\underset{j=0}{\sum }}\overset{k}{\underset{i=1}{\sum }}\mu_{j}\cdot max\left[\left(q_{j}-1\right)\left(y_{t+i}-{\hat{y}}_{t+i}^{q_{j}}\right),\;q_{j}\left(y_{t+i}-{\hat{y}}_{t+i}^{q_{j}}\right)\right]$$

(3) $$MSELoss\;\left(t\right)=\frac{1}{k}\sum_{i=1}^{k}{\left(y_{t+i}-{\hat{y}}_{t+i}\right)}^{2}$$

The CWQLoss includes a learnable weight ($\mu_{j})$ for each quantile prediction (Equation (2)). These weights are called quantile weights and are learned end-to-end by gradient descent along with the rest of the network weights. Quantile weights can be learned with or without additional restrictions imposed on their values. In case of restrictions, the quantile weights are also learned end-to-end similarly to the nonconstrained case, but their values are constrained in the following way (Section 4.3 shows the beneficial impact of these restrictions): (a) An odd number of quantile forecasts with the middle quantile being always the median, that is, the set Q of quantile’s probabilities satisfies the following restrictions: $Q={\left\{q_{j}\right\}}_{j=0}^{2m}$ with $q_{i}>q_{j}$ for i>j, and $q_{m}=0.5.$ (b) The elements in Q are mirror-*symmetrical* around the median $\left(q_{m}\right)$, that is: $q_{j}$ *=* $1-q_{2m-j}$. (c) The learnable weights ($\mu_{j})$ are mirror *identical* around the middle weight ($\mu_{m}$), that is: $\mu_{j}$ *=* $\mu_{2m-j}$. (d) The learnable weights ($\mu_{j})$ are normalized, such that $\sum \mu_{j}=1$. This normalization is done with a softmax activation function (Figure 9). Imposing these constraints is important to obtain a working model, both to have an accurate point forecast and to have adequate quantile forecasts. In particular, the last constraint is necessary to stabilize the learning phase and facilitate proper convergence of the learning algorithm. Experimental results (Section 4) show that not imposing this constraint results in a high crossover of the quantile forecasts and difficulties for the convergence of the loss function. Quantile forecast crossover occurs when a quantile forecast for a quantile with an associated lower probability is greater than the forecast for an upper probability quantile. An interesting observation is that, after training, the weights ($\mu_{j}$) always have the same pattern with a larger value for the middle weight ($\mu_{m})\;$and smaller and similar values for the other weights ($\mu_{j\neq m}$). Interestingly, in the effort to reduce the quantile loss (CWQLoss), the gradient descent minimization algorithm always reinforces the value of the weight associated with the median quantile forecast. 

In Figure 9, the entries to the CWQLoss are: (a) the ground-truth targets, which are part of the training dataset (${\left\{y_{t+i}\right\}}_{i=1\ldots k}$), (b) the M quantiles forecast produced by the model (${\left\{{\hat{y}}_{t+i}^{q_{j}}\right\}}_{i=1\ldots k}^{j=0\ldots 2m}$), (c) the set of quantile probabilities (Q), which is a hyperparameter of the model and, (d) the loss constrained weights (${\left\{\mu_{j}\right\}}_{j=0}^{m}$), which are included as trainable variables in the computational graph of the model, using a deep learning framework [68], thus allowing to be trained end-to-end by gradient descent simultaneously with the rest of the network weights. It is interesting to note that it was unnecessary to create a smooth version of the pinball loss as defined in Equation (1), also mentioned in [44]. The definition of the pinball loss in Equation (1) is similar to other loss or activation functions that include a maximum operator, e.g., ReLU, max-margin (linear SVM). These functions also do not require a smoothing process for proper gradient descent operation. In fact, applying Huber or log-cosh smoothing to Equation (1) produces an undesirable effect of quantile collapse towards the median.

It is important to note that the training of the CWQFNN model is performed end-to-end from scratch for the entire network (the base model and the 2m+1 final FC layers). The process for selecting the base model can be based on its point forecast performance, but once the base model is selected, it is included in the CWQFNN architecture with randomly initialized weights. Therefore, the CWQFNN model is a unique single-shot trained model and not a two-stage trained model where the base model is pre-trained in a previous training stage.

In this work, all the models for quantile forecasting (CWQFNN architecture with different base models) use the loss function in Equation (2) and the other models based on neural networks that perform only point forecasts (models in Figure 6, Figure 7 and Figure 8) use the mean square error (MSE) loss function (Equation (3)).

In Section 4.3, the impact of the constraints on quantile-weights is discussed, presenting what the usual values taken by the quantile-weights after training are. The importance of these weights in the quality of the forecasts is also evaluated.

The NN that produces the point forecasts in Figure 9 is called the base model. The selection of the base model has an impact on prediction performance, and when the base model is an additive ensemble NN, we achieve the best performance (Section 4). The only requirement for a base model is to be trainable end-to-end by gradient descent and support the addition of a final layer in both the training and prediction stages. Any of the models included in Figure 6 and Figure 8 can serve as a base model. We do not consider sequence-to-sequence (Seq2seq) models as a base model since the forward pass for the training, and test stages are different, which creates added complexity for the proposed extension of M final layers. This is the same reason why the Seq2seq model is not included as a learning block in AE architectures.

As a summary, the steps to implementing the algorithm to train a CWQFNN architecture are:

1. Obtain the training and validation sets using a sliding window method (Figure 1);
2. Select a base model (Figure 5, Figure 6, Figure 7 and Figure 8);
3. Select the quantile probabilities used in the forecasts ($i.e.,Q={\left\{q_{j}\right\}}_{j=0}^{2m}$) (Figure 9)
4. Apply the selected base model within the CWQFNN architecture (Figure 9);
5. The output of the CWQFNN model will be 2m+1 (i.e., |Q|) quantile forecasts for each of the k time-ahead predictions, along with the learned quantile weights (${\left\{\mu_{j}\right\}}_{j=0}^{m}$) applied in the CWQLoss (Equation (2)).

We implemented all the neural network models (CWQFNN, deep learning, Seq2seq, attention and additive ensemble) in python using Tensorflow/Keras [68]. For all other models, we used the scikit-learn python package [69]. All computations have been performed on a commercial PC (i7–4720-HQ, 16 GB RAM).

To tune the network weights, we have used mini-batch gradient descent with early-stopping as an implicit regularization mechanism and the best solution search. We have applied early stopping using the validation set to choose the best configuration. Early stopping is based on computing the validation loss (in our case, CWQLoss) at the end of each epoch. If the validation loss at the end of a certain number of previous epochs does not obtain any reduction, the training process stops, and the weights corresponding to the best validation loss are used as final weights. The waiting period (number of previous epochs used to compare any decrease in the validation loss) is called the patience value. As a summary of the training parameters for the neural networks in this work: (a) We have used Adam as the optimization method. The parameters used are α (learning rate): 0.001, $\beta_{1}$: 0.9, $\beta_{2}$: 0.999 and ε: 1 × 10^−8^, which are the default values proposed in [70]. (b) We have used mini-batch gradient descent with a batch size of 10, using 150 epochs for training with early stopping and 10 epochs as patience value.

**Figure 10:**
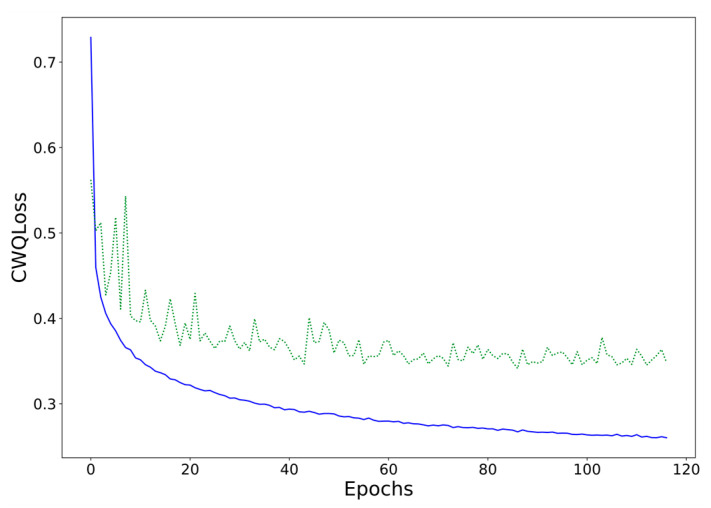
Evolution of CWQLoss for the training (solid-blue line) and validation (dotted-green line) sets for successive epochs during training. Using a CWQFNN architecture based on an additive ensemble model.

It may also be of interest to investigate the evolution of the loss function (CWQLoss) during training. Figure 10 shows this evolution for a CWQFNN architecture with a base model consisting of an additive ensemble with 5 blocks and 4 fully connected layers per block. We can see how the training converges quite smoothly after 20–30 epochs with some initial noise. This behavior has been observed in most models, with some models being more difficult to train, such as the Seq2seq and the larger ensemble models.

## 4. Results

In this section, we present in detail the point forecast performance metrics obtained by all the models considered for this research and the probabilistic forecast metrics for CWQFNN with different base models. An additional aim is to present (under homogeneous evaluation criteria) the results obtained by classic forecasting methods together with new or less used methods, e.g., deep learning ensembles, DMD, deep learning models with combinations of CNN and LSTM layers and the proposed CWQFNN architecture.

The analysis of results is based on the following models: (a) classic ML (random forest and linear regression) [18,19,20,21]; (b) dynamic mode decomposition (DMD) [22,23,24]; (c) Seq2seq models [27,28,29]; (d) deep learning models based on recurrent and convolutional layers [19,20]; (e) additive ensemble deep learning models [9]; and (f) CWQFNN architectures. As mentioned in Section 3.2, all models based on the CWQFNN architecture use the loss function: CWQLoss (Equation (2)) (Figure 9), and the other models, based on neural networks (models in Figure 6, Figure 7 and Figure 8), use the mean square error (MSE) loss function (Equation (3)). These latter models only perform point forecasts based on the MSE loss, while the CWQFNN models perform two types of forecast: (a) probabilistic forecast (based on CWQLoss) and (b) point forecast by adopting the forecast for the median (0.5 quantiles) as the point forecast. In all these cases, the optimization of the loss function is performed with gradient descent. The classic ML and DMD models employ specific optimization methods that are not based on gradient descent.

All the results presented are based on the dataset described in Section 3.1 using exclusively the test sets described in that section. Since the load values were scaled in the range [0–1], we have inversely scaled the predicted values to calculate all point forecast metrics. This is important because the original range of values of the predicted magnitude is [7370–39550] with an average value of 21,478.2, which means that the metrics that refer to absolute errors (not rates) are tremendously impacted depending on the scale factor. For probabilistic forecast metrics, we have opted to calculate them with scaled outputs. The probabilistic metrics related to quantile probabilities focus on the estimated error that the output signal remains within a certain cutoff value and that estimate is not altered by the scale (e.g., absolute average coverage error). Additionally, probabilistic metrics related to the width of central prediction intervals (e.g., sharpness, Winkler scores) when normalized are more easily interpreted as a proportion within a normalized set of output values.

All the models are applied to a multivariate multi-output regression problem with a k-step ahead forecasting horizon of 24, 168 or 720 load values (24, 168 or 720 h ahead) using different numbers of past time-slots (24, 168 and 720) that corresponds to consider as predictors the past day, week or month (in hours). Additionally, we have studied the results considering different feature lengths for each predictor: a single scalar value (the past electric load) or a vector of values (the past predicted load plus date/time variables with a one-hot encoding as categorical values). Considering the terminology proposed in Section 3.1, we use the symbols k for the number of forecast values, p for the number of predictor time-slots and f for the length of features used as predictors.

All results for the CWQFNN architecture have been obtained with different base models, but all share the same set Q of quantile’s probabilities and all applying the quantile weights constraints presented in Section 3.2. The set Q used is: Q = [0.01,0.25,0.5,0.75,0.99] for all CWQFNN models.

### 4.1. Point Forecasts

To perform the comparison between models, we have used several point forecast metrics: median absolute error (MAD), relative root mean square error (RRMSE) and symmetric mean absolute percentage error (sMAPE). The definition of the point forecast performance metrics are herewith presented, where Y corresponds to the ground-truth values, $\hat{Y}$ are the predicted values, $\bar{Y}$ is the mean values of Y, $Y_{i}$ is each particular ground-truth value, ${\hat{Y}}_{i}\;$is each particular predicted value, and *N* is the number of samples in the test set (Section 3.1):

(4) $$MAD=Median\left(\left|Y-\hat{Y}\right|\right)$$

(5) $$RRMSE=\frac{\sqrt{\sum_{i=1}^{N}{\left(Y_{i}-{\hat{Y}}_{i}\;\right)}^{2}}}{\sqrt{\sum_{i=1}^{N}{\left(\;Y_{i}\;\right)}^{2}}}$$

(6) $$sMAPE=\frac{100}{N}\overset{N}{\underset{i=1}{\sum }}2\frac{\left|Y_{i}-{\hat{Y}}_{i}\right|}{\left|Y_{i}\right|+\left|{\hat{Y}}_{i}\right|}\;\%$$

**Figure 11:**
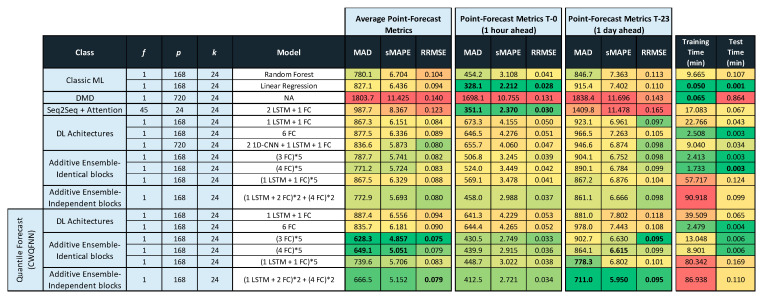
Point forecast performance metrics for a forecast time horizon of 24 h (k = 24) for the first (T-0) and last (T-23) hour forecast and average forecast over the 24 h (1 day) time horizon. Results for a selection of best models for each group of models. Table is color-coded (column-wise) with a green–red palette corresponding to best-worst results, respectively. The best two values per column are highlighted in bold.

**Figure 12:**
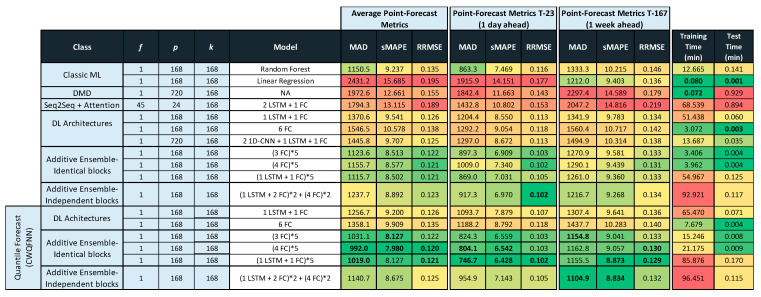
Point forecast performance metrics for a forecast time horizon of 168 h (k = 168) for the 23rd (T-23) and last (T-167) hour forecast and average forecast over the 168-h (1 week) time horizon. Results for a selection of best models for each group of models. Table is color-coded (column-wise) with a green–red palette corresponding to best-worst results, respectively. The best two values per column are highlighted in bold.

**Figure 13:**
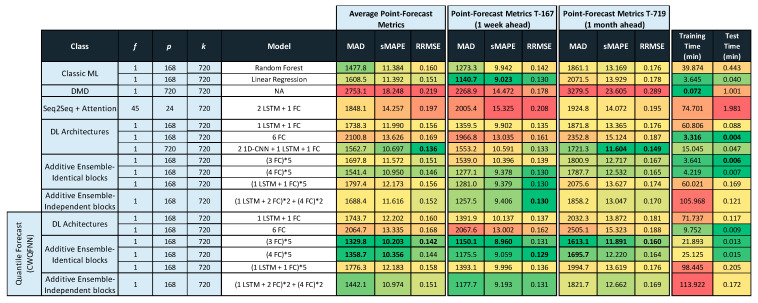
Point forecast performance metrics for a forecast time horizon of 720 h (k = 720) for the 167th (T-167) and last (T-719) hour forecast and average forecast over the 720-h (1 month) time horizon. Results for a selection of best models for each group of models. Table is color-coded (column-wise) with a green–red palette corresponding to best-worst results, respectively. The best two values per column are highlighted in bold.

The metrics in Equations (4)–(6) provide a separate value for each of the k predictions in the forecast horizon. The forecast metrics for some selected time-ahead predictions (e.g., 1 h, 1-day, 1-week, 1-month) are given separately in the following tables of results together with an average for all the k predictions in the forecast horizon (Figure 11, Figure 12 and Figure 13). All these metrics have values greater than zero with no upper limit, except sMAPE, which has an upper limit of 200%. In all cases, the smaller the value, the better the result. The metrics MAD, sMAPE and RRMSE, are error metrics. They are always positive, with a value of zero corresponding to the best result. The RRMSE and SMAPE will be considered important since they calculate the ratio between the prediction error and a value related to the actual quantity to be predicted.

Tables in Figure 11, Figure 12 and Figure 13 provide the main point forecast results for the most representative models of each model type (Section 3.2). Tables also include the training and prediction times using the training and test sets of the selected dataset (Section 3.1). The complete set of results for all model configurations are included in Figure A1 (Appendix A) for a forecast time horizon of 24 h (k = 24). From the results in Figure A1 are extracted the most important results presented in Figure 11. The models selected in Figure 11 have been used in Figure 12 and Figure 13 for forecast time horizons (k) of 168 and 720, respectively. Tables are color-coded (column-wise) with a green–red palette corresponding to best-worst results. In addition, the best two values per column are highlighted in bold.

Figure 11 provides point forecast performance metrics for a forecast time horizon of 24 h (k = 24) for the first (T-0) and last (T-23) hour forecast and average forecast over the 24 h (1 day) time horizon. Figure 12 provides point forecast performance metrics for a forecast time horizon of 168 h (k = 168) for the 23rd (T-23) and last (T-167) hour forecast and average forecast over the 168-h (1 week) time horizon. Figure 13 provides point forecast performance metrics for a forecast time horizon of 720 h (k = 720) for the 167th (T-167) and last (T-719) hour forecast and average forecast over the 720-h (1 month) time horizon. Tables include the time required to perform training of the models and prediction (using the test set) in minutes. Figure 11, Figure 12 and Figure 13 present the forecast metrics obtained under different scenarios (Section 3.2) and considering different combinations of values for the parameters f, *p* and *k*. The range of values for these parameters are the ones explained in Section 3.1.

In addition to providing the point forecast metrics for all models for k = 24, Appendix A A provides supplemental details for Figure 12 and Figure 13 by giving the forecast metrics at additional intermediate time-slots, thus Figure A2 (Appendix A) extends Figure 12 with an additional forecast at T-0 (first hour), and similarly Figure A3 (Appendix A) extends Figure 13 with two additional forecasts at T-0 (1 h) and T-23 (1-day).

In all tables in Figure 11, Figure 12 and Figure 13, the description of the models includes the number and type of layers used: CNN, LSTM, and fully connected layers (FC), forming a sequence separated by the + sign, e.g., 2 LSTM + 1FC indicates a model with two LSTM layers followed by one FC layer. The additive ensemble (AE) models have been divided into AE-identical blocks and AE-independent blocks (Section 3.2). The description of AE-identical blocks is formed by repeating blocks where the configuration of each repeating block is included in parenthesis with an asterisk and a number to the right of the asterisk that indicates the number of repetitions of the block, e.g., (4 FC) * 5 indicates a model with five identical blocks each composed of four FC layers. When blocks share weights, it is represented by the string WS at the end of the description. The description of AE-independent blocks is formed by different blocks with a possibly different architecture and different types of inputs per block. In those cases where there are different types of input per block, we have marked that in tables by several values of f separated by a backslash (\). The different blocks in an AE-independent blocks architecture are represented as a sequence of repeating blocks separated by a + sign, e.g., (1 LSTM + 3 FC)*2 + (4 FC)*5 indicates a model with two different blocks where the first is repeated two times and is composed by one LSTM and three FC layers, and the second is repeated five times and is composed by four FC layers.

All results for the CWQFNN architecture have been obtained with different base models, but all share the same set Q of quantile’s probabilities and all apply the quantile weights constraints presented in Section 3.2. The set Q used is: Q = [0.01,0.25,0.5,0.75,0.99] for all CWQFNN models. Section 4.3 presents in detail the impact of changing the number of quantiles and, more important, the constraints imposed on the quantile weights. The results are more or less independent of the number of quantiles as far as this number is neither very high nor very low (e.g., only the median), but the impact of the imposed restrictions is really important and, without constraints, the point forecast metrics are extremely poor.

Results in Figure 11, Figure 12 and Figure 13 provide the following interesting conclusions:

(a). DMD models do not provide the best results under any configuration.
(b). Classic ML models (linear regression) provide the best results for very short-term forecasts considering the maximum forecast time horizon in each scenario. For example, linear regression provides best results at T-0 for a forecast time-horizon of 24 h, the same happens for a time horizon of 168 h (Figure A2 in Appendix A), and is among the best result models for T-0, T-23 and T-167 for a time horizon of 720 h (Figure A3 in Appendix A).
  It is important to note that the ML models do not produce a multi-output prediction. Therefore, it is necessary to create a specific predictor for each predicted value. This is the reason for the good behavior of these models in short-term forecasts. The interesting point is that for long-term forecasts, a single DL, AE, or CWQFNN model can produce better forecasts than many ML models, each trained on a specific expected output. A possible explanation for this behavior is that the further the forecast is in time, the relationship between predictors and outputs is less linear, and the correlation between outputs is more relevant.
(c). Seq2seq+ attention gives better results than Seq2seq. They present excellent results for very short-term forecasts and poor results for average and longer-term forecasts. Seq2seq models only have results for a value of f equal to 45. For a value of f equal to 1, the network had difficulties converging, and the results were poor (Figure A1 in Appendix A). The combination of CNN and LSTM layers provides poor results for Seq2seq models, while pure recurring networks with one or two LSTM layers provide the best results.
(d). DL models provide good average performance metrics. The best models are simple combinations of LSTM and FC layers (e.g., 1 LSTM + 1 FC), sequences of a few FC layers (e.g., 6 FC), and simple combinations of 1D-CNN and LSTM layers (e.g., 2 1D-CNN+ 1 LSTM + 1 FC). The architectures with 2D-CNN layers provide poor results.
(e). Additive ensemble (AE) deep learning models are excellent performance architectures for long-term forecasting and average results. There is not much difference in performance between the AE architecture with Identical and Independent blocks, but considering its greater simplicity, we can justify that the Identical blocks architecture is the one that offers the best results. AE models perform best with blocks composed of a few FC layers repeated a small number of times (e.g., (3 FC)*5) and simple combinations of LSTM and FC layers also repeated a small number of times (e.g., (1 LSTM + 1 FC)*5).
  The good behavior of the AE deep learning models is related to a better exploration in the solution space due to the independent random initialization of each block in the ensemble [36,37]. This explanation better justifies the good behavior that the ability of AE models to reduce overfitting given that all the regularization techniques used (drop-out, batch normalization, weight sharing) do not provide any performance improvement (Figure A1 in Appendix A), which indicates that the focus of this problem should not be on overfitting but on obtaining a sufficiently rich network structure that adjusts to the characteristics of the data.
(f). CWQFNN architectures present the best results for average and long-term forecasting for almost all point forecast metrics. To achieve these results, the best base model is an AE-Identical blocks architecture with a small number of repeating blocks formed by a few FC layers (e.g., (3 FC)*5 or (4 FC)*5). The good behavior of this architecture is maintained for all forecast time horizons (k = 24, 168, 720).

The median forecast of a CWQFNN architecture with a specific base model produces better results than the same stand-alone base model, i.e., not forming part of a CWQFNN architecture. It is important to analyze the possible reasons why a model that produces good prediction results improves by being part of a CWQFNN architecture as a base model. It is worth noting that this improvement is highly dependent on applying the correct weights to each of the pinball losses associated with each quantile (Section 4.3).

**Figure 14:**
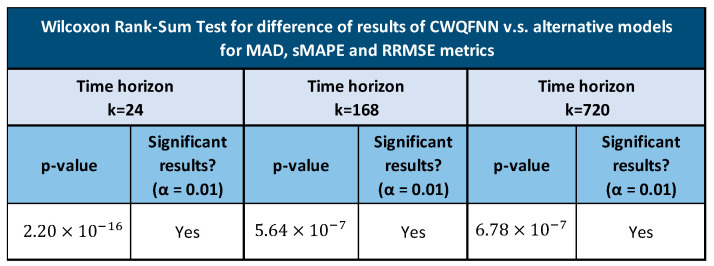
Results of the Wilcoxon rank-sum test to check the significance of the better results obtained by CWQFNN vs. non-quantile forecast models.

To ensure that the results obtained for the CWQFNN architecture are better (from a statistical point of view) than those obtained by not using it, we have applied the Wilcoxon paired one-sided rank-sum test for the comparison of performance metrics between the CWQFNN architecture with the base model: (3 FC)*5 (Figure 11, Figure 12 and Figure 13) and the rest of non-quantile forecast models (ML, DMD, Seq2seq, DL and AE) for the MAD, sMAPE and RRMSE metrics. Figure 14 presents the results for applying the hypothesis test considering different forecast time horizon scenarios (k = 24,168 and 720). The *p*-value indicates if the results allow (or not) to reject the null hypothesis that is associated with a non-significant difference in the results. The test used is one-side to specifically check if the group of alternative models has a higher or equal ranked mean than the best model. From the results in Figure 14, using a significance level (*α*) of 1%, we conclude that the point forecast metrics obtained with the best CWQFNN model are significantly better than the alternative non-quantile models.

### 4.2. Probabilistic Forecasts

Several probabilistic forecast metrics [71] will be used to evaluate the performance of quantile forecasts: Quantile score (QS), Crossover rate score (CORS), absolute average coverage error (AACE), Winkler score (WS) [72] and sharpness. The metrics AACE, WS and sharpness, are provided for two central prediction intervals (PI) with associated probabilities (1−α)×100% of 50% and 98%.

The definition of these metrics is as follows, where (a) N is the number of samples in the tests set (Section 3.1); (b) (1−α) is the probability associated with a PI; (c) α is the probability outside a PI; (d) $L_{t,s}^{\alpha }$ and $U_{t,s}^{\alpha }$ are the lower and upper quantile forecast for a (1−α)% PI for the sample at time t for the s-time ahead prediction. $L_{t,s}^{\alpha }$ corresponds to the quantile forecast for the quantile (α/2) and $U_{t,s}^{\alpha }$ corresponds to the quantile forecast for the quantile (1−α/2); (e) $\delta_{t,s}^{\alpha }$ is the forecasted PI width, i.e., $\delta_{t,s}^{\alpha }=U_{t,s}^{\alpha }-L_{t,s}^{\alpha }$. (f) The PI nominal confidence (PINC) is by definition equal to (1−α); (g) ${\hat{y}}_{t+s}^{q_{i}}$ is the quantile forecast for the quantile of probability $q_{i}$ of the sample at time t for the s-time ahead prediction, where $q_{i}$ is a monotonically increasing list of probabilities indexed by i, i.e., $q_{i}>q_{j}$ for i>j.

The QS metric (Equation (8)) is an average metric for all samples in the test set of an unweighted version of the CWQLoss (Equation (7)):

(7) $$Unweighted\_CWQLoss\;\left(t\right)=\frac{1}{k\cdot \left(2m+1\right)}\overset{2m}{\underset{j=0}{\sum }}\overset{k}{\underset{i=1}{\sum }}max\left[\left(q_{j}-1\right)\left(y_{t+i}-{\hat{y}}_{t+i}^{q_{j}}\right),\;q_{j}\left(y_{t+i}-{\hat{y}}_{t+i}^{q_{j}}\right)\right]$$

(8) $$Quantile\;Score\;\left(QS\right)=\frac{1}{N}\sum_{i=1}^{N}Unweighted\_CWQLoss\;\left(i\right)$$

The Winkler score metric (Equation (10)) is an average of penalized forecasted PI widths. No penalty is applied if the real value is within the forecasted PI; otherwise, we add a penalty equal to the ratio of the distance from the forecast value to the upper or lower PI boundary (the one closest to the forecast value) and α/2. An error in a wide PI interval (small α) is more penalized:

(9) $$WS\;\left(t,s,\alpha \right)=\{\begin{array}{c} \delta_{t,s}^{\alpha }\;L_{t,s}^{\alpha }\;\leq y_{t+s}\leq U_{t,s}^{\alpha }\;\\ \delta_{t,s}^{\alpha }+2\left(L_{t,s}^{\alpha }-y_{t+s}\right)/\alpha \;y_{t+s}<L_{t,s}^{\alpha }\;\\ \delta_{t,s}^{\alpha }+2\left(y_{t+s}-U_{t,s}^{\alpha }\right)/\alpha \;y_{t+s}>U_{t,s}^{\alpha }\; \end{array}$$

(10) $$WS\;\left(\alpha \right)=\frac{1}{N\cdot k}\sum_{i=1}^{N}\sum_{j=1}^{k}WS\;\left(i,j,\alpha \right)$$

The sharpness metric (Equation (11)) is an average of the forecasted PI widths:

(11) $$Sharpness\;\left(\alpha \right)=\frac{1}{N\cdot k}\sum_{i=1}^{N}\sum_{j=1}^{k}\delta_{i,j}^{\alpha }$$

The AACE metric (Equation (14)) is an estimate of the difference between the expected and actual PI nominal confidence value. It indicates the error between the expected proportion of points within a PI, i.e., (1−α) and the empirical value:

(12) $$Coverage\;Indicator\;\left(t,s,\alpha \right)=COInd\left(t,s,\alpha \right)=\{\begin{array}{c} 1\;L_{t,s}^{\alpha }\;\leq y_{t+s}\leq U_{t,s}^{\alpha }\;\\ 0\;Not\left(L_{t,s}^{\alpha }\;\leq y_{t+s}\leq U_{t,s}^{\alpha }\right) \end{array}$$

(13) $$PI\;Coverage\;Probability\;\left(\alpha \right)=PICP\left(\alpha \right)=\frac{1}{N\cdot k}\sum_{i=1}^{N}\sum_{j=1}^{k}COInd\left(i,j,\alpha \right)$$

(14) AACE(α)=|PICP(α)−PINC(α)|=|PICP(α)−(1−α)|

The CORS metric (Equation (16)) is the probability that a crossover will occur between any of the quantile forecasts made for all quantiles, all test samples, and all *k*-ahead forecast values. This crossover metric is stricter than alternative crossover metrics that only consider crossovers between quantile forecasts at the boundaries of a PI interval. The metric defined in Equation (15) considers crossover for any quantile pair. The crossover indicator function will mark any noncompliance if the quantile forecasts for consecutive quantile probabilities do not follow a strictly increasing sequence:

(15) $$CrossOver\;Indicator\left(t,s\right)=CRInd\left(t,s\right)=\{\begin{array}{c} 1\;if\;{\hat{y}}_{t+s}^{q_{i}}>{\hat{y}}_{t+s}^{q_{j}}\;for\;some\;i<j\;\\ 0\;if\;{\hat{y}}_{t+s}^{q_{i}}<{\hat{y}}_{t+s}^{q_{j}}\;for\;all\;i<j \end{array}$$

(16) $$CORS=\frac{1}{N\cdot k}\overset{N}{\underset{i=1}{\sum }}\overset{k}{\underset{j=1}{\sum }}CRInd\left(i,j\right)$$

All the above probabilistic metrics are error metrics where a lower value indicates a better result. The QS metric has unbounded positive values. WS and sharpness have unbounded values, but negative values are only applicable to difunctional models with high crossover. CORS and AACE have values in the range [0–1]. Some of the probabilistic metrics are interval metrics associated with a PI (WS, AACE and sharpness) that consider only a particular interval produced by a pair of quantile forecasts corresponding to the upper and lower limits of the PI; while others are quantile metrics (QS and CORS) that consider all the quantiles produced by the model.

**Figure 15:**
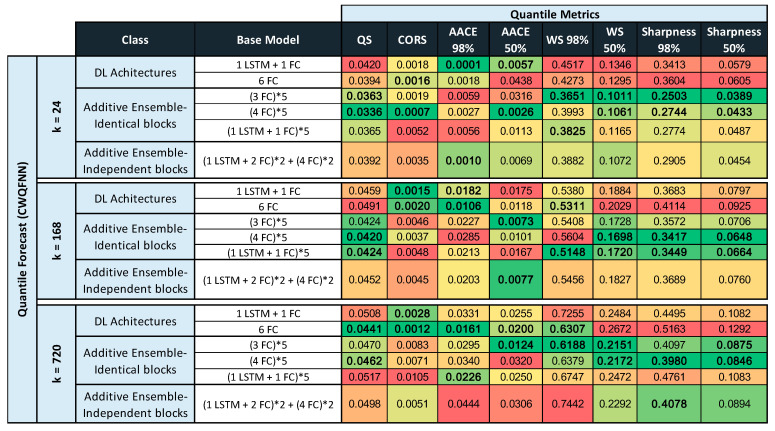
Probabilistic forecast performance metrics for the CWQFNN architecture with different DL and AE base models for different forecast time horizons (k). Considering the metrics: quantile score (QS), crossover rate score (CORS), absolute average coverage error (AACE), Winkler score (WS) and sharpness. AACE, WS and sharpness are provided for two central prediction intervals with associated probabilities of 50% and 98%. Table is color-coded (column-wise) with a green–red palette corresponding to best-worst results, respectively. The best two values per column are highlighted in bold. The assignment of color/bold has been carried out separately for each of the three blocks in the figure (*k* = 24, 168 and 720).

The probabilistic forecast metrics for the CWQFNN models are given in Figure 15 for the metrics: QS, CORS, AACE, WS and sharpness. The metrics AACE, WS and sharpness, are provided for two central prediction intervals (PI) with associated probabilities (1−α)% of 50% and 98%. Three forecast time horizons are considered (k = 24,168 and 720). All results are for: f = 1 and p = 168 (Section 3.1). The best results for the different probabilistic forecast metrics are more or less concentrated on the base models with additive ensemble-identical blocks (3 FC)*5 and (4 FC)*5, but are more evenly distributed among all models than the best results for the point forecast metrics, which are clearly concentrated on these two models. The metrics indicate excellent results with a probability of crossover between 0.07% and 0.12%, a sharpness at 98% PI between 0.25 and 0.4 for a [0–1] range of output values, and an AACE (error between expected and actual PI nominal confidence value) at 98% PI between 0.01% and 2.26%. In all cases, quantile forecast metrics worsen with higher forecast time horizons (k), as expected.

**Figure 16:**
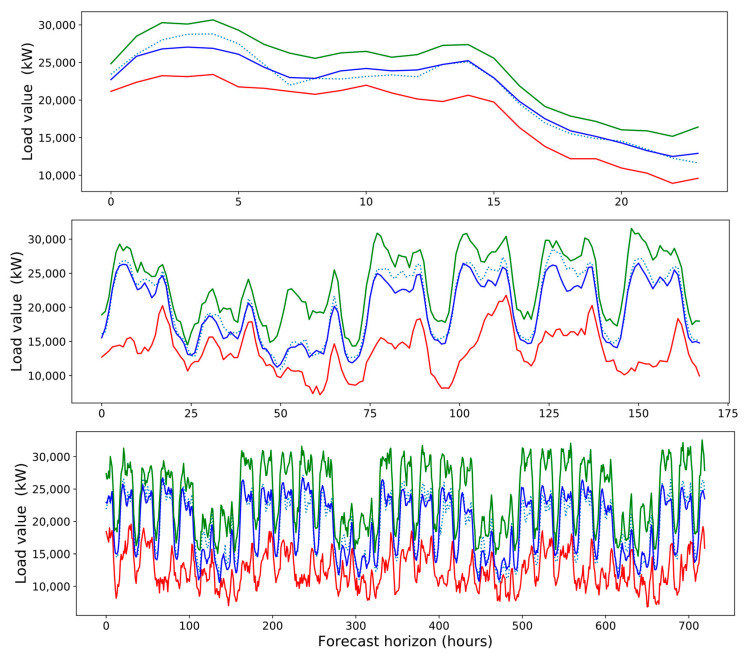
Load forecasts (kilowatts) for different forecast time horizons: 24 (**upper**), 168 (**middle**) and 720 h (**lower** chart), showing four signals: ground-truth load (dotted line), 0.99 quantile forecast (green line), median (blue line) and 0.01 quantile forecast (red line) (model: CWQFNN-[(4 FC)*5] (f  = 1, p = 168)).

To provide a visual indication of the quality of the probabilistic forecasts at different forecast time horizons, Figure 16 shows a comparison between real (ground-truth) load signals and their forecasts as we increase the forecast time horizon. The different diagrams are 24/168/720 h time windows taken at random points in the test set. Four load signals are shown: Ground-truth load (dotted blue line), 0.99 quantile forecast (green line), median (blue line) and 0.01 quantile forecast (red line). The 0.99 and 0.01 quantile forecasts serve as the boundary values for a central PI of 98% probability. All charts share the same model: CWQFNN-[(4 FC)*5] (f = 1, p = 168)). The median forecast is taken as the point forecast of the model. The point forecast signal is close to the real one most of the time and always within the central PI of 98% probability. In almost all cases, the point forecast signal follows the real one and produces a smoothed version of it.

### 4.3. Impact of Quantile-Weights Restrictions

**Figure 17:**
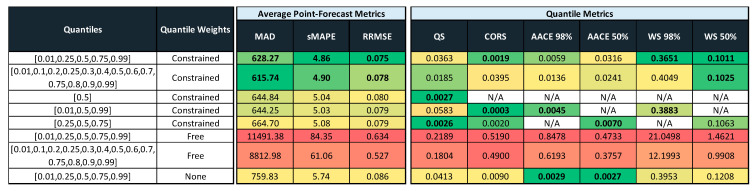
Point and quantile forecast performance metrics for CWQFNN with the same base model, but considering different values for the following configuration parameters: (a) number and value of quantiles, (b) type of quantile-weights with possible values of constrained quantile-weights (constrained), nonconstrained quantile-weights (free), and no quantile-weights at all (none) (model: CWQFNN-[(3 FC)*5] (f = 1, p = 168, k = 24)). Table is color-coded (column-wise) with a green–red palette corresponding to best-worst results, respectively. The best two values per column are highlighted in bold.

Figure 17 provides the point and quantile forecast performance metrics for a CWQFNN architecture with the same base model (model: CWQFNN-[(3 FC)*5] (f = 1, p = 168, k = 24)), but considering different values for the number of quantiles (Q), and the inclusion or not of restrictions on the quantile weights ($\mu_{j}$ in Equation (2)).

In case of no restrictions, the quantile weights are learned end-to-end along with the rest of the network weights without any restrictions (marked as free in Figure 17). In case of restrictions, the quantile weights are also learned end-to-end similarly to the previous case, but their values are constrained to being mirror identical around the middleweight and with the quantile probabilities being mirror symmetrical around the median (Section 3.2) (marked as constrained in Figure 17). The results are more or less independent of the number of quantiles as far as this number is neither very high nor very low (e.g., only the median). However, the impact of the constraints is really significant, and without constraints, the point forecast metrics are extremely bad. Without constraints, the crossover rate (CORS) is extremely high, and all PI metrics (AACE, WS and sharpness) have very bad values. Finally, the results for the configuration without quantile weights are given at the end of the table (marked as none in Figure 17); in this case, we can observe how the point forecasts are worse than the configurations, including the restricted weights, while the probabilistic metrics are also worse, but not so impacted.

It is interesting to examine the final values (after training) of the quantile weights with and without constraints. In the case of constrained weights, some typical values for the weights are μ = [0.106, 0.120, 0.548, 0.120, 0.106]; that is, the weight corresponding to the median is higher than the rest of the weights, which are smaller (but not negligible) and of similar value. While a typical list of weights (after training) for the unconstrained case is μ = [1.00, 2.70 × 10^−12^, 3.12 × 10^−13^, 1.14 × 10^−12^, 3.23 × 10^−12^]; that is, the optimization process focuses only on optimizing one of the quantiles, missing the real objective of the algorithm of training a single model to have multiple-quantile forecasts.

As a summary, Figure 17 compares the results obtained by a CWQFNN with the same base model while changing the number of quantiles and the type of constraints on the quantile weights.

### 4.4. Time Evolution of Point and Probabilistic Forecasts

**Figure 18:**
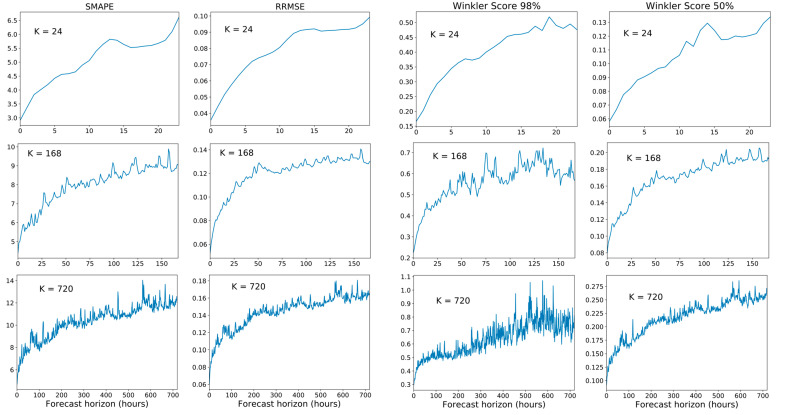
Evolution of the point forecast metrics (sMAPE and RRMSE) (**left** charts) and probabilistic forecast metrics (Winkler score at 98% and 50%) (**right** charts) for successive 1 h time intervals for different forecast time horizons (k = 24,168,720) for the CWQFNN architecture with base model [(4 FC)*5] (f = 1, p = 168).

It is interesting to analyze the evolution of the point and probabilistic forecast metrics for each time slot in a series of future forecasts (Figure 18). This evolution depends on the forecasting model, ranging from almost linear to exponential and-with-and without intermediate/final plateaus. In all cases, the metric values have a noisy, monotonically increasing (worst) behavior. Figure 18 shows the sMAPE, and RRMSE point-forecast metrics (left charts) and the Winkler score probabilistic forecast metrics for a PI with probabilities 98% and 50% (right charts) for successive predicted time-slots for the CWQFNN architecture with base model [(4 FC)*5] (f = 1, p = 168). It is interesting how the time evolution patterns between point and probabilistic metrics share similarities. It is important to note that the point forecast for the CWQFNN models is made by taking the quantile forecast for the 0.5 quantiles, explaining the similarities found.

**Figure 19:**
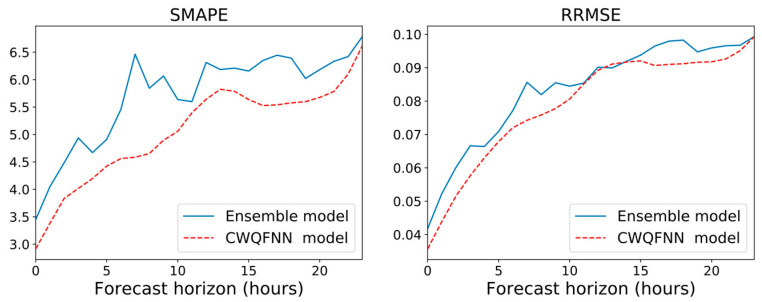
Comparison of the evolution of two point-forecast metrics (sMAPE and RRMSE) for successive 1 h time intervals in a forecast time horizon of 24 h for the same additive ensemble model ([(4 FC)*5] (f = 1, p = 168)) when incorporated into a CWQFNN architecture as its base model (dotted red line) vs. as a stand-alone AE model (solid blue line).

Figure 19 shows a comparison of the evolution of two point-forecast metrics (sMAPE and RRMSE) for successive 1 h time intervals in a forecast time horizon of 24 h between a stand-alone AE model (solid blue line) and the same model as part of a CWQFNN architecture (dotted red line). We observe how the metrics for the CWQFNN architecture are almost always smaller than its non-quantile forecast counterpart, and additionally, the time evolution is more linear and gradual.

### 4.5. Influence of the Sliding-Window Length

**Figure 20:**
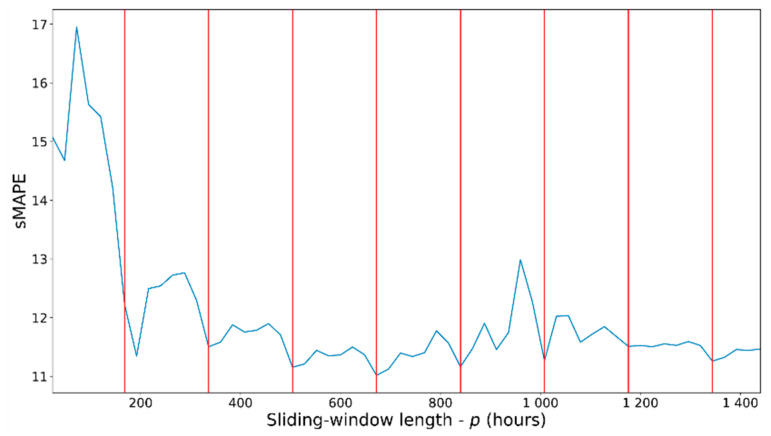
Evolution of the forecast metric (sMAPE) vs. the value of parameter p (sliding window length in hours), obtained with the DMD model. Successive vertical red lines are 1 week apart. The maximum decrease in error occurs with a sliding window of about one week (p= 168).

A different evolution of the forecast metrics can be studied by changing the parameter p (length of the sliding window in hours) or the number of previous time-steps used as predictors. Figure 20 presents this evolution for all the performance metrics with a sliding window length ranging from 24 to 1440 h, i.e., from 1 day to 60 previous days. In Figure 20, each 1 week (168 h) interval is marked with a vertical red line. We can see how an increase in performance is obtained by increasing the value of p up to a value of 168 (1 week of sliding window length), after which the increase is much smaller and even decreases with a p value greater than 720 (4 weeks). The ascending and descending periodic metric values in each weekly interval are also interesting. The results in Figure 20 can explain the reason why most of the best models are achieved with a p value between 168 and 720, except for the Seq2seq models for which there is a balance between the greater information contained in longer predictor sequences and the difficulty in training a longer time-series, even using an LSTM.

## 5. Discussion

The main contribution of this research is to propose a novel quantile neural network architecture (CWQFNN) with a new quantile loss. This new quantile loss imposes a constrained weighted version of the pinball loss, providing several advantages as demonstrated by the results (Section 4). In Figure 17, we can see how models with no restrictions on the quantile weights (free weights) obtain extremely poor results, while models with restricted weights obtain the best results. A possible explanatory resource for this behavior can be found in the results obtained in multitask learning [73] as a way to improve the performance prediction for classification/regression and the fact that the CWQLoss implicitly applies to multitask learning approach. It is also interesting the connection between multitasking and multiobjective learning provided in [74] with a proposal for an upper bound for the multitask loss that can be optimized by gradient descent with constrained weights per task, but this connection needs further investigation.

Interestingly, the median forecast of a CWQFNN architecture with a specific base model provides better results than the same base model as a stand-alone model. It is important to analyze the possible reasons why a model that produces good prediction results improves by being part of a CWQFNN architecture as a base model. This improvement is highly dependent on applying the correct weights to each of the pinball losses associated with each quantile (Section 4.3). Therefore, it seems again that adding weights to the pinball loss improves results, but it is equally important to provide a certain structure to those weights to guarantee the improvement. In addition, we observe in Figure 19 how the error metrics for the CWQFNN architecture are almost always smaller than its non-quantile forecast counterpart and additionally, the time evolution is more linear and gradual. This may justify CWQFNN’s better average performance and better performance in long-term forecasts.

In Figure 11, Figure 12, Figure 13 and Figure 14, we see the point forecast results for CWQFNN compared with alternative models. CWQFNN presents the best results for average and long-term forecasting for almost all point forecast metrics. These results are obtained with a CWQFNN architecture with a base model consisting of an additive ensemble-identical blocks architecture with a small number of repeating blocks formed by a few FC layers (e.g., (3 FC)*5 or (4 FC)*5). The good behavior of this architecture is maintained for all forecast time horizons (k = 24, 168, 720). Likewise, the best results for the different probabilistic forecast metrics (Figure 15) are more or less concentrated on the same base models (additive ensemble-identical blocks-(3 FC)*5 and (4 FC)*5) but are more evenly distributed among all models than the best results for the point forecast metrics, which are clearly concentrated on these two models.

It is important to mention the good behavior of the additive ensemble deep learning models, which produce many of the best results for stand-alone models and most of the best results when being part of a CWQFNN architecture. This behavior is obtained both in point and probabilistic forecast scenarios.

In summary, we can conclude that the proposed architecture is a promising model for probabilistic time-series forecasting. The added complexity introduced by the new loss function (CWQLoss) is largely compensated by the improved performance achieved in point and probabilistic forecasts.

## 6. Conclusions

This work presents a novel quantile forecasting architecture (CWQFNN) that extends a point forecast NN by transforming it into a multi-quantile forecasting model. The underlying NN is called the base model and must be end-to-end trainable by gradient descent and support the addition of a final layer in both the training and prediction stages. The CWQFNN architecture also proposes a novel quantile loss (CWQLoss) based on the pinball loss. CWQLoss incorporates specifically defined constrained weights associated with each quantile. The constrained parameters are learned along with the rest of the network weights by gradient descent. We show the importance of the added weights and the defined constraints for the new quantile loss.

The proposed architecture provides excellent point and quantile forecast performance metrics and is applied to short and medium-term load forecasting (SMTLF) on a dataset of real power consumption from a medium-sized city in Spain. An extensive analysis of results is provided, comparing the results obtained by CWQFNN against an extensive list of alternative classic and state-of-the-art machine learning forecasting models and considering the influence of important model parameters.

The proposed architecture (CWQFNN) does not require transforming the base model or adding complex extensions to the loss function to ensure efficient quantile forecasts with a small crossover rate. The experimental results allow us to conclude that the CWQFNN architecture presents the best forecast performance metrics for average and long-term forecasting and achieves its best performance when the base model is an additive ensemble deep learning model.

As the future line of research related to the present work, we plan to further investigate the connections between the performance improvement obtained by applying the CWQLoss, which implicitly applies a multitask learning approach [73], with the direction taken by works that address the multitask learning as a multiobjective optimization problem [74,75] as a potential resource to explain the behavior shown by the CWQFNN architecture and the impact of, including the constrained weights into the pinball loss. A possible extension to this investigation could be to apply a multiobjective loss [74] to quantile forecasting in SMTLF.

## Data Availability

Restrictions apply to the availability of these data. Data was obtained from Iberdrola and are available from the authors with the permission of Iberdrola.

## Appendix A

This appendix presents the full set of results, from which the most important results and conclusions have been drawn.

Note: The following figure has been divided into subfigures to facilitate its formatting, but they are part of a single figure (Figure A1).
